# Relationship between quadriceps muscle computed tomography measurement and motor function, muscle mass, and sarcopenia diagnosis

**DOI:** 10.3389/fendo.2023.1259350

**Published:** 2023-11-16

**Authors:** Takafumi Mizuno, Yasumoto Matsui, Makiko Tomida, Yasuo Suzuki, Shinya Ishizuka, Tsuyoshi Watanabe, Marie Takemura, Yukiko Nishita, Chikako Tange, Hiroshi Shimokata, Shiro Imagama, Rei Otsuka, Hidenori Arai

**Affiliations:** ^1^Department of Orthopaedic Surgery, Nagoya University Graduate School of Medicine, Nagoya, Japan; ^2^Center for Frailty and Locomotive Syndrome, National Center for Geriatrics and GerontologyObu, Obu, Japan; ^3^Department of Epidemiology of Aging, National Center for Geriatrics and Gerontology, Obu, Japan; ^4^Graduate School of Humanities and Social Sciences, Nagoya City University, Nagoya, Japan; ^5^Faculty of Health Sciences, Department of Human Care Engineering, Nihon Fukushi University, Mihama, Japan; ^6^Graduate School of Nutritional Sciences, Nagoya University of Arts and Sciences, Nisshin, Japan; ^7^National Center for Geriatrics and Gerontology, Obu, Japan

**Keywords:** computed tomography, quadriceps femoris, muscle quality, muscle mass, sarcopenia

## Abstract

**Background:**

The quadriceps muscle is one of the human body’s largest and most clinically important muscles and is evaluated using mid-thigh computed tomography (CT); however, its relationship with motor function and sarcopenia remains unclear. Herein, we investigated the relationship between the cross-sectional area (CSA) of the quadriceps muscle, CT attenuation value (CTV), dual-energy X-ray absorptiometry muscle mass measurements, and muscle strength and motor function to evaluate the relationship between muscle mass loss and motor function decline, determine the diagnostic ability for sarcopenia, and confirm the usefulness of quadriceps muscle CT evaluation.

**Methods:**

A total of 472 middle-aged and older community dwellers (254 men and 218 women) aged ≥40 years (mean age: 62.3 years) were included in this study. The quantity and quality of the quadriceps muscle were assessed using CSA and CTV (CSA×CTV) as a composite index multiplied by quality and quantity. Age-adjusted partial correlations by sex with eight motor functions (knee extension muscle strength, power, normal walking speed, fast walking speed, grip strength, sit-up ability, balance ability, and reaction time) were evaluated, including correction methods for height, weight, and body mass index (BMI). Further, the accuracy of sarcopenia diagnosis was evaluated using appendicular muscle mass with dual-energy X-ray absorptiometry measurements, grip strength, and walking speed as the gold standard, and receiver operating characteristic curves were plotted to evaluate diagnostic performance.

**Results:**

In men, CSA and CSA×CTV were significantly associated with seven of the eight motor functions (p<0.05), excluding only balance ability. BMI-corrected CSA was significantly correlated with all eight motor functions in men and women (p<0.05). In the diagnosis of sarcopenia based on skeletal muscle index, CSA (area under the curve (AUC) 0.935) and CSA×CTV (AUC 0.936) and their correction by height (CSA/height (AUC 0.917) and CSA×CTV/height (AUC 0.920)) were highly accurate and useful for diagnosis in men but moderately accurate in women (CSA (AUC 0.809), CSA×CTV (AUC 0.824), CSA/height (AUC 0.799), CSA×CTV/height (AUC 0.814)).

**Conclusion:**

The present results showed that a single CT image of the quadriceps muscle at the mid-thigh is useful for diagnosing sarcopenic changes, such as loss of muscle mass, muscle weakness, and muscle function.

## Introduction

Sarcopenia is a critical condition that increases the risk of mortality and functional disability in older individuals (1). In 2018, the European Working Group on Sarcopenia in Older People focused on muscle mass and quality as key parameters for diagnosing sarcopenia (2). Imaging modalities such as computed tomography (CT), magnetic resonance imaging, and ultrasonography enable a detailed evaluation of the constituent elements of the transverse section and have been reported to be potentially useful in assessing muscle quality (3–7). CT could be a better form of assessment because of its accuracy, reproducibility, and objectivity (8). Prior studies using CT have attempted to assess muscle mass using the cross-sectional area (CSA) and muscle quality using the CT attenuation value (CTV) at the mid-thigh or abdomen (4, 9). However, which muscles should be evaluated to assess muscle mass and quality and how the results should be corrected remain unclear. Skeletal muscle mass index (SMI) is an index used in the diagnostic criteria for sarcopenia, usually calculated by dividing skeletal muscle mass (ASM) by the square of height. On the other hand, as recently reported, there is a debate regarding the superiority of ASM alone or ASM/body mass index (BMI) over SMI in predicting prognosis (10–12), this traditional method is considered a controversial correction method. Therefore, it is necessary to verify which method of muscle mass index is appropriate: uncorrected, based on height, height squared, body weight, or BMI.

We previously reported that the percentage of CSA reduction in the quadriceps (Qc) muscle was greater than that in the hamstrings on mid-thigh CT of older individuals (13). Further, because the Qc muscle is one of the largest muscles, it may be particularly susceptible to the effects of aging and sarcopenia (14) (15). Additionally, the Qc muscle is relatively easy to assess using portable devices, such as ultrasound scanners (3) and is therefore of clinical importance. Although we previously reported data from a hospital cohort (16), there have been few detailed evaluations of the relationship between Qc CT and other modalities on muscle mass and physical function in community populations, and no reports have yet clarified the diagnostic ability of Qc CT for muscle mass or strength loss or physical function decline. Herein, we investigated the relationship between Qc CT measurements, including various corrections, and dual-energy X-ray absorptiometry (DXA) measurements, including various corrections, muscle strength, and motor function, in a cross-sectional survey of middle-aged and older individuals in the general Japanese population to confirm the usefulness of Qc CT by evaluating its diagnostic ability for sarcopenia and by exploring its relationship with muscle mass loss and impaired motor function.

## Methods

### Participants

The National Institute for Longevity Sciences-Longitudinal Study of Aging (NILS-LSA) investigated age-related differences among randomly selected middle-aged and older community dwellers aged ≥40 years from resident registrations in Obu city and Higashiura town (Aichi Prefecture, Japan). This study observed and documented the normal aging process in individuals over time, and was operated as a dynamic cohort comprising age- and sex-matched random participants; for dropouts under 80 years of age at follow-up, new participants of the same sex and age group were recruited, as well as new participants aged ≥40 years were recruited annually (17). The seventh wave of the NILS-LSA was conducted from July 2010 to July 2012 and included 2,330 participants. Among these, 525 consecutive participants were included in this study after February 2, 2012. The right mid-thigh CT data of the participants were stored in Digital Imaging and Communications in Medicine format with measurable CTVs. Five of the 525 participants who refused or could not undergo CT imaging or who had poor-quality or difficult-to-evaluate images, as well as 48 participants in whom motor function could not be tested owing to illness on the day of the study (e.g., knee pain, back pain, fatigue, lightheadedness), schedule delays, or severe comorbidities were excluded. Finally, 472 participants (254 men and 218 women, mean age: 62.3 years, age range: 40–89 years) in whom the right knee extension muscle strength (KES) could be measured were included in the analysis. Furthermore, 367 (135 men and 232 women, mean age: 77.2 ± 7.1 years, age range: 53–96 years) out of 500 patients who visited the Integrated Healthy Aging Clinic at the National Center for Geriatrics and Gerontology, Japan, from 2016 to 2019 were included for external validation in this study (18, 19). Of these, patients with gait impairment due to severe knee or hip osteoarthritis, and progressive motor disease such as Parkinson’s disease were excluded because of the difficulty in accurately assessing physical function, and cases with missing data were excluded. This research was approved by the institutional ethics committee (certification no. 1361 and 881) and was conducted in accordance with the ethical standards laid down in the 1964 Declaration of Helsinki and its later amendments. Written informed consent was obtained from all participants.

### Qc femoris CT

**Figure 1:**
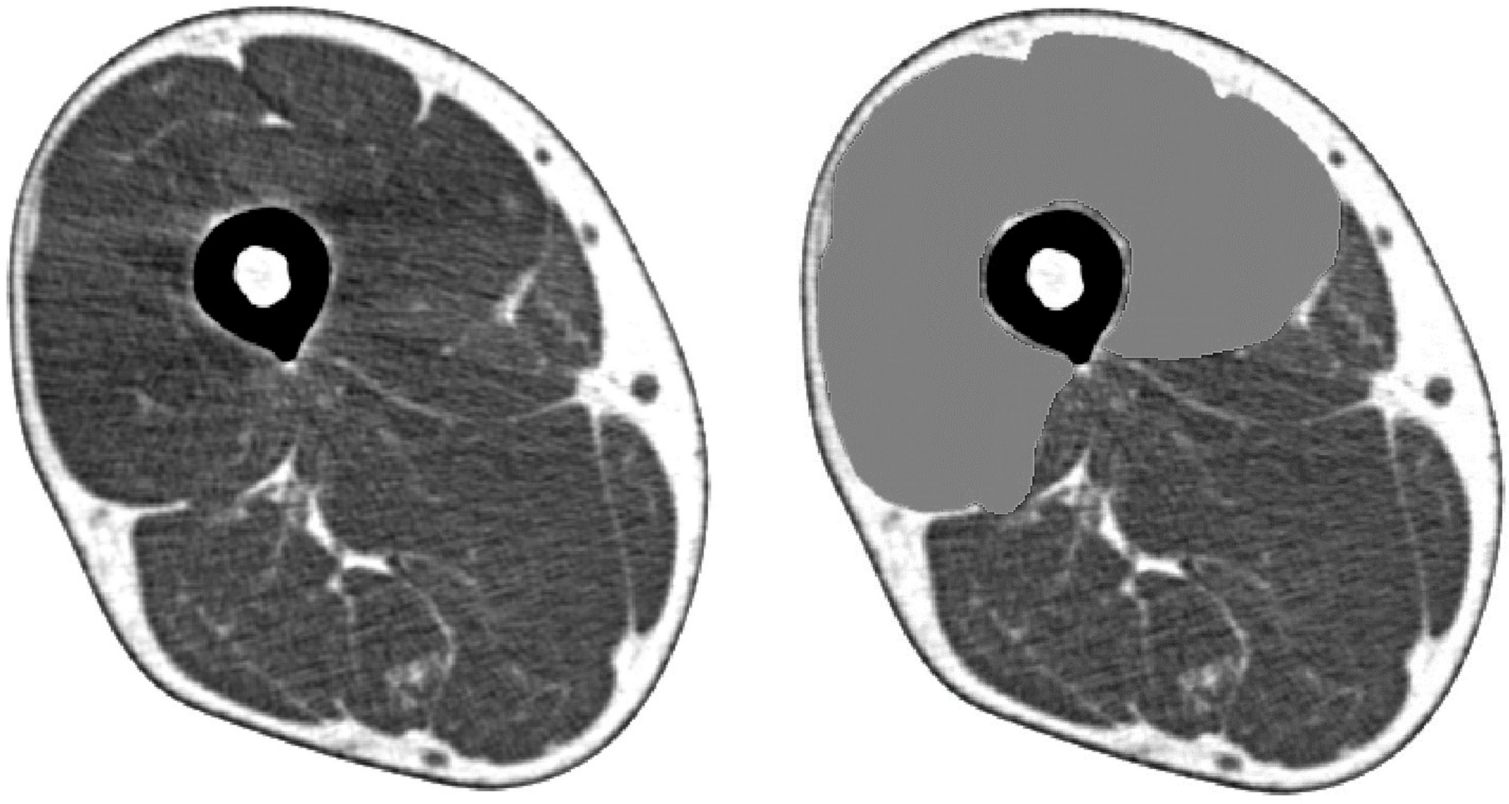
Cross-sectional muscle area of the quadriceps femoris determined by mid-thigh computed tomography.

Participants underwent a single-slice CT examination of the right mid-thigh (location: midpoint of the superior pole of the patella and inguinal crease; settings: 120 kV, 120 mA; rotation time: 1 s; field of view: 233 mm) using SOMATOM Sensation 64™ (Siemens, Munich, Germany). CSAs and CTVs from the CT images were analyzed using sliceOmatic software version 5.0 (TomoVision, Magog, Canada). CSAs were evaluated as a measure of the total Qc muscle, including the intermuscular fat mass, and CTVs were evaluated as a measure of muscle quality, reflecting the intramuscular fat and intramyocellular lipid levels. Manual range selection was used for the Qc muscle (**Figure 1**). Additionally, CSA multiplied by CTV was defined as a composite index multiplied by quality and quantity. The following parameters were created to evaluate the correction method: CSA/height, CSA/height^2^, CSA/weight, CSA/BMI, CTV/BMI, CSA×CTV/height, CSA×CTV/height^2^, CSA×CTV/weight, CSA×CTV/BMI. The SOMATOM Sensation 64 (Siemens, Munich, Germany; settings: 120 kV, 120 mA; rotation time: 1 s; field of view: 233 mm) and Aquilion CXL (CANON, Tochigi, Japan; settings: 135 kV, 150 mA; rotation time: 1 s; field of view: 4.0 mm) were used in the external validation cohort using the same analysis methods.

### Skeletal muscle mass measured using DXA

Skeletal muscle mass was measured using QDR-4500 DXA (Hologic, Bedford, MA, USA). The participants were placed supine on the DXA table, with their limbs held close to their bodies. The whole-body lean soft tissue mass comprised the soft tissue masses of the arms, legs, and trunk. The ASM was determined by combining the lean soft tissue masses of the arms and legs, and the SMI (kg/m^2^) was calculated as ASM/height^2^. Given the controversy of this method, the ASM, SMI, ASM/weight, and ASM/BMI were additionally used in the analysis (2). Another DXA machine (Lunar iDXA, GE Healthcare, Chicago, IL, USA) was used in the external validation cohort.

### Motor function measurement

#### KES and power

KES was measured using T.K.K.1281 (Takei, Niigata, Japan). Participants were instructed to sit on a chair and extend both knees from a 90° flexed position, and the highest value of three tests conducted on the right lower extremity was used. The value of the extension muscle strength divided by the total area of the Qc muscle (KES/CSA, N/cm^2^) was also used as one of the muscle quality indices. This was modified from a previous study, which defined knee extension power as the total lean muscle mass divided by the total leg area (N/cm^3^) (20).

Leg extension power was measured using T.K.K.4236 (Takei, Niigata, Japan). The participants were fastened to a chair with a seat belt and instructed to extend their knees as quickly and strongly as possible in a horizontal direction with their feet placed on a foot plate, and the maximum value from eight attempts was used. KES in the external validation cohort was measured using ZP-500N (Imada Co., Ltd., Toyohashi, Aichi, Japan) (21).

### Measurement of physical performance

Grip strength (kg) was measured using T.K.K.4301a (Takei, Niigata, Japan). Walking speed was measured using the YW-3 walking analysis system (Yagami, Aichi, Japan); normal and fast walking speeds were determined over a 10-m walk. Regarding balance ability, closed-eye balance (seconds) was measured using T.K.K.4315a (Takei, Niigata, Japan), with both hands on the waist, one foot on the ground, eyes closed at the point of balance, and posture maintained. The maximum value of two measurements (a maximum of 180 seconds) was used. The whole-body reaction time (seconds) was used as an index of agility; using T.K.K.4312a (Takei, Niigata, Japan), the average value of five repetitions was taken as the adopted value (i.e., legs shoulder-width apart on the measurement mat, knees lightly flexed in preparation, and a quick jump when the lamp lit up red). Upper body raising (times), an index of endurance described as a sit-up, was measured using T.K.K. 4329a (Takei, Niigata, Japan). The participants were assessed while lying supine on the seat with their knees and ankles draped over a cushion in front of the abdominal table and with their hands folded behind their heads, performing as many sit-ups as possible. The number of times per measurement (30 seconds) was used as the adopted value. In the external validation cohort, grip strength was measured using the ZP-500N force gauge (IMADA Co., Ltd., Toyohashi, Aichi, Japan), while normal walking speed was measured using Walkway MW-1000 (ANIMA Corp., Tokyo, Japan).

### Other parameters

The height and weight of all participants were assessed, and their BMI was calculated as weight/height^2^ (kg/m^2^). The participants’ medical histories (including hypertension, dyslipidemia, diabetes, heart disease, and stroke), vitamin administration, and smoking status were obtained through questionnaires.

### Statistical analysis

Statistical analyses were performed using SAS version 9.3 (SAS Institute, NC, USA), with statistical significance set at p<0.05. Measurements were not excluded, even if they were outliers. CSA, ASM, and their corrected values divided by height, height^2^, weight, and BMI were used as indices of muscle mass. CTV and its corrected value divided by BMI and KES/CSA were used as indices of muscle quality. CSA×CTV and its corrected value divided by height, height^2^, weight, and BMI were used as combined indices of muscle mass and muscle quality. The relationship between these variables and physical function was evaluated according to sex using Pearson’s correlation coefficient, adjusted for age. For reference, a test of differences in correlation coefficients (22) was performed for the SMI. Additionally, the Asian Working Group for Sarcopenia (AWGS) 2019 reference values were used to assess muscle function loss (grip strength <28 kg for men and <18 kg for women and/or walking speed 1.0 m/second) and muscle mass loss (DXA <7.0 kg/m^2^ for men and <5.4 kg/m^2^ for women) (23). Those who fulfilled these categories were also classified as having sarcopenia (muscle mass loss and reduced physical function). Receiver operating characteristic (ROC) curves were plotted to evaluate the diagnostic performance. The diagnostic accuracy was evaluated based on the area under the curve (AUC), with 0.9–1.0, 0.7–0.9, and 0.5–0.7 being considered high, moderate, and low, respectively. A comparison of ROC curves was also carried out for grip strength and SMI for reference (24). The optimal cutoff value was determined as the value closest to the upper left of the ROC curve, and sensitivity and specificity at that value were calculated. Diagnostic performance was validated in the external validation cohort using the determined cutoff values. Diagnostic odds ratio (DOR) was calculated with 0.5 added to all contingency tables because calculating it is impossible when the number of false positives or false negatives is 0 (25, 26).

## Results

### Participant characteristics

The characteristics of the participants are summarized in **Table 1**.

**Table 1:** Characteristics of the study participants.

	Men		Women		
	n=254		n=218		p-value
Age, years	62.4	±10.2	62.1	±10.4	0.760
Body height, cm	166.2	±6.4	154.1	±5.9	<0.001
Body weight, kg	64.2	±8.6	53.4	±9.7	<0.001
BMI, kg/m^2^	23.2	±2.6	22.5	±3.6	0.007
**Medical history**					
Stroke, n (%)	5	(2.0)	8	(3.7)	0.276
Heart disease, n (%)	21	(8.3)	6	(2.8)	0.010
Diabetes, n (%)	26	(10.2)	13	(6.0)	0.097
Hypertension, n (%)	93	(36.6)	60	(27.5)	0.039
Dyslipidemia, n (%)	50	(19.7)	57	(26.1)	0.099
Smoker, n (%)	54	(21.3)	7	(3.2)	<0.001
Vitamin D administration, n (%)	2	(0.8)	7	(3.2)	0.088
**Sarcopenia diagnosis**					
Muscle mass loss (%)	68	(26.8)	39	(18.1)	0.027
Muscle function loss (%)	25	(9.8)	31	(14.4)	0.154
Sarcopenia (%)	14	(5.5)	11	(5.1)	1.000
**Quantity**					
CSA, cm^2^	58.0	±9.5	42.3	±7.4	<0.001
CSA / height, cm	0.349	±0.054	0.274	±0.044	<0.001
CSA / height^2^, %	0.210	±0.033	0.178	±0.027	<0.001
CSA / weight, cm^2^/kg	0.907	±0.117	0.800	±0.119	<0.001
CSA / BMI, 10^4^·cm^4^/kg	2.51	±0.36	1.90	±0.32	<0.001
ASM, kg	20.8	±2.8	14.4	±2.3	<0.001
SMI, kg / m^2^	7.53	±0.77	6.06	±0.76	<0.001
ASM / weight, %	32.5	±2.3	27.2	±2.4	<0.001
ASM / BMI, m^2^	0.90	±0.10	0.65	±0.08	<0.001
**Quality**					
CTV, HU	54.3	±3.4	50.6	±4.1	<0.001
CTV / BMI, HU·m^2^/kg	2.37	±0.34	2.31	±0.41	0.075
KES / CSA, N/cm^2^	7.81	±1.40	6.96	±1.32	<0.001
**Quality×Quantity**					
CSA × CTV, cm^2^·HU	3157	±595	2146	±444	<0.001
CSA × CTV / height, cm·HU	19.0	±3.4	13.9	±2.7	<0.001
CSA × CTV / height^2^, HU	0.114	±0.020	0.090	±0.017	<0.001
CSA × CTV / weight, cm^2^·HU/kg	49.4	±8.2	40.7	±8.0	<0.001
CSA × CTV / BMI, 10^4^·cm^4^·HU/kg	137	±25	97	±21	<0.001
**Physical performance**					
Grip, kg	37.6	±6.5	23.3	±4.5	<0.001
KES, N	453	±108	294	±76	<0.001
Leg Power, W	535	±156	321	±93	<0.001
Sit up, times	13.4	±5.0	8.0	±5.5	<0.001
Walking, m/seconds	1.41	±0.18	1.38	±0.16	0.032
Fast walking, m/seconds	1.92	±0.24	1.80	±0.20	<0.001
Balance, seconds	16.1	±24.0	13.9	±19.5	0.292
Reaction, seconds	0.43	±0.08	0.46	±0.07	<0.001

Values are expressed as numbers (%).

Means are expressed ± standard deviation.

p-values were obtained from the t-test for continuous data and the χ^2^ test and fisher's exact test for categorical data.

Abbreviations: BMI, body mass index; CSA, cross-sectional area; ASM, appendicular skeletal muscle mass; SMI, skeletal muscle mass index; CTV, computed tomography attenuation value; HU, Hounsfield unit; KES, knee extension strength.

### Physical function and CT and DXA measurements

Non-adjusted correlations were significantly related for most variables (**Tables S1**, **S2**). **Tables 2**, **3** show the results for age-adjusted partial correlations by sex. The following sections concisely describe the results; please refer to the Tables for correlation coefficients and other information.

**Table 2:** Correlation of physical functions with computed tomography and dual-energy X-ray absorptiometry measurement (1).

Men	KES r- value	n=	254p-value	vs. SMIp-value	Leg powerr-value	n=	245p-value	vs. SMIp-value	Walkingr-value	n=	254p-value	vs. SMIp-value	Fast walkingr-value	n=	254p-value	vs. SMIp-value
**Quantity**																
CSA	**0.523**		**<0.001**	0.109	**0.422**	*	**<0.001**	**0.049**	**0.174**		**0.005**	0.331	**0.171**		**0.007**	0.628
CSA / height	**0.526**		**<0.001**	0.097	**0.355**		**<0.001**	0.270	**0.175**		**0.005**	0.328	**0.168**		**0.007**	0.651
CSA / height^2^	**0.498**		**<0.001**	0.216	**0.270**		**<0.001**	0.946	**0.165**		**0.009**	0.388	**0.156**		**0.013**	0.757
CSA / weight	**0.365**		**<0.001**	0.545	**0.154**		**0.016**	0.204	**0.182**		**0.004**	0.291	**0.173**		**0.006**	0.610
CSA / BMI	**0.381**		**<0.001**	0.691	**0.331**		**<0.001**	0.422	**0.196**		**0.002**	0.221	**0.192**		**0.002**	0.468
ASM	**0.352**		**<0.001**	0.438	**0.385**		**<0.001**	0.138	0.085		0.176	0.966	**0.129**		**0.040**	0.993
SMI	**0.411**		**<0.001**	–	**0.265**		**<0.001**	–	0.089		0.158	–	**0.129**		**0.041**	–
ASM / weight	**0.171**		**0.006**	**0.003**	0.081		0.208	**0.037**	0.081		0.198	0.928	**0.142**		**0.024**	0.877
ASM / BMI	**0.125**		**0.047**	**<0.001**	**0.254**		**<0.001**	0.897	0.075		0.238	0.869	**0.125**		**0.048**	0.964
**Quantity**																
CTV	**0.172**		**0.006**	**0.003**	0.088		0.171	**0.044**	**0.211**		**<0.001**	0.163	0.103		0.101	0.775
CTV / BMI	**-0.159**		**0.011**	**0.002**	-0.120		0.062	0.097	0.051		0.423	0.665	0.010		0.874	0.182
KES / CSA	**0.708**	*	**<0.001**	**<0.001**	**0.249**		**<0.001**	0.853	0.117		0.063	0.751	0.076		0.229	0.551
**Quality× Quantity**																
CSA × CTV	**0.545**		**<0.001**	0.051	**0.420**		**<0.001**	0.052	**0.227**		**<0.001**	0.112	**0.190**		**0.002**	0.480
CSA × CTV / height	**0.549**	*	**<0.001**	**0.044**	**0.359**		**<0.001**	0.250	**0.229**		**<0.001**	0.108	**0.188**		**0.003**	0.493
CSA × CTV / height^2^	**0.524**		**<0.001**	0.103	**0.281**		**<0.001**	0.844	**0.218**		**<0.001**	0.138	**0.177**		**0.005**	0.578
CSA × CTV / weight	**0.357**		**<0.001**	0.481	**0.151**		**0.018**	0.192	**0.215**		**<0.001**	0.148	**0.172**		**0.006**	0.616
CSA × CTV / BMI	**0.363**	** **	**<0.001**	0.529	**0.290**	** **	**<0.001**	0.767	**0.223**	** **	**<0.001**	0.123	**0.183**	** **	**0.004**	0.531
Women	KES r- value	n=	217p-value	vs. SMIp-value	Leg powerr-value	n=	204p-value	vs. SMIp-value	Walkingr-value	n=	216p-value	vs. SMIp-value	Fast walkingr-value	n=	254p-value	vs. SMIp-value
**Quantity**																
CSA	**0.606**	*	**<0.001**	**0.001**	**0.365**		**<0.001**	0.169	0.065		0.341	0.717	0.084		0.220	0.743
CSA / height	**0.60**	*	**<0.001**	**0.001**	**0.338**		**<0.001**	0.287	0.034		0.625	0.972	0.053		0.443	0.998
CSA / height^2^	**0.570**	*	**<0.001**	**0.005**	**0.293**		**<0.001**	0.575	-0.003		0.964	0.780	0.016		0.819	0.704
CSA / weight	**0.311**		**<0.001**	0.583	0.105		0.136	0.160	0.092		0.177	0.519	**0.167**		**0.015**	0.233
CSA / BMI	**0.362**		**<0.001**	0.964	**0.191**		**0.006**	0.598	**0.160**		**0.019**	0.176	**0.233**		**<0.001**	0.057
ASM	**0.375**		**<0.001**	0.834	**0.302**		**<0.001**	0.507	0.096		0.163	0.498	0.029		0.673	0.808
SMI	**0.358**		**<0.001**	–	**0.241**		**0.001**	–	0.030		0.660	–	-0.053		0.445	–
ASM / weight	0.103		0.133	**0.005**	0.067		0.341	0.074	**0.198**		**0.004**	0.079	**0.175**		**0.010**	0.201
ASM / BMI	**0.166**		**0.015**	**0.032**	**0.173**		**0.014**	0.474	**0.244**	*	**<0.001**	**0.024**	**0.231**		**<0.001**	0.060
**Quality**																
CTV	**0.162**		**0.018**	**0.029**	0.071		0.315	0.080	-0.005		0.946	0.792	0.090		0.189	0.697
CTV / BMI	**-0.177**		**0.009**	**0.043**	**-0.141**		**0.044**	0.300	0.068		0.322	0.697	**0.143**		**0.037**	0.347
KES / CSA	**0.738**	*	**<0.001**	**<0.001**	**0.307**		**<0.001**	0.476	**0.166**		**0.015**	0.157	**0.273**	*	**<0.001**	**0.019**
**Quality× Quantity**																
CSA × CTV	**0.622**	*	**<0.001**	**<0.001**	**0.367**		**<0.001**	0.164	0.051		0.455	0.828	0.112		0.104	0.540
CSA × CTV / height	**0.611**	*	**<0.001**	**<0.001**	**0.338**		**<0.001**	0.289	0.023		0.735	0.943	0.085		0.215	0.736
CSA × CTV / height^2^	**0.574**	*	**<0.001**	**0.004**	**0.295**		**<0.001**	0.563	-0.007		0.914	0.814	0.054		0.434	0.990
CSA × CTV / weight	**0.301**		**<0.001**	0.511	0.100		0.155	0.145	0.069		0.313	0.687	**0.164**		**0.016**	0.244
CSA × CTV / BMI	**0.350**	** **	**<0.001**	0.931	**0.174**	** **	**0.013**	0.482	0.125	** **	0.068	0.327	**0.220**	** **	**0.001**	0.078

Partial correlation: adjusted for age.

Bold: p<0.05.

* is significantly greater in absolute value than the correlation coefficient between SMI and the same physical function in a test of difference of correlations (p<0.05).

Abbreviations: CSA, cross-sectional area; BMI, body mass index; ASM, appendicular skeletal muscle mass; SMI, skeletal muscle mass index; CTV, computed tomography attenuation value; KES, knee extension strength.

**Table 3:** Correlation of physical functions with computed tomography and dual-energy X-ray absorptiometry measurement (2).

Men	Gripr-value	n=	254p-value	vs. SMIp-value	Sit upr-value	n=	249p-value	vs. SMIp-value	Balancer-value	n=	254p-value	vs. SMIp-value	Reactionr-value	n=	253p-value	vs. SMIp-value
**Quantity**																
CSA	**0.374**		**<0.001**	0.813	**0.179**		**0.005**	0.911	0.064		0.314	0.568	**-0.138**		**0.029**	0.345
CSA / height	**0.283**		**<0.001**	0.366	**0.211**		**<0.001**	0.631	0.090		0.152	0.383	**-0.164**		**0.009**	0.214
CSA / height^2^	**0.179**		**0.004**	**0.033**	**0.230**		**<0.001**	0.481	0.110		0.080	0.272	**-0.179**		**0.004**	0.157
CSA / weight	0.033		0.596	**<0.001**	**0.329**		**<0.001**	0.058	**0.222**	*	**<0.001**	**0.017**	**-0.184**		**0.003**	0.141
CSA / BMI	**0.280**		**<0.001**	0.347	**0.264**		**<0.001**	0.266	**0.167**		**0.008**	0.080	**-0.134**		**0.033**	0.367
ASM	**0.519**	*	**<0.001**	**0.022**	0.080		0.208	0.317	-0.070		0.265	0.518	0.006		0.920	0.592
SMI	**0.355**		**<0.001**	–	**0.169**		**0.008**	–	-0.013		0.840	–	-0.054		0.391	–
ASM / weight	**0.223**		**<0.001**	0.104	**0.329**		**<0.001**	0.058	0.117		0.064	0.241	-0.029		0.649	0.775
ASM / BMI	**0.437**		**<0.001**	0.275	**0.146**		**0.021**	0.797	0.006		0.927	0.938	0.038		0.544	0.859
**Quality**																
CTV	0.090		0.154	**0.002**	**0.250**		**<0.001**	0.346	0.105		0.096	0.299	**-0.134**		**0.033**	0.366
CTV / BMI	-0.113		0.073	**0.004**	0.120		0.060	0.577	0.102		0.107	0.317	-0.033		0.597	0.815
KES / CSA	**0.148**		**0.019**	**0.013**	**0.166**		**0.009**	0.975	0.077		0.219	0.467	-0.118		0.061	0.471
**Quality× Quantity**																
CSA × CTV	**0.367**		**<0.001**	0.878	**0.242**		**<0.001**	0.395	0.102		0.104	0.313	**-0.167**		**0.008**	0.203
CSA × CTV / height	**0.284**		**<0.001**	0.373	**0.274**		**<0.001**	0.219	**0.129**		**0.041**	0.192	**-0.191**		**0.002**	0.119
CSA × CTV / height^2^	**0.189**		**0.003**	**0.043**	**0.292**		**<0.001**	0.150	**0.147**		**0.019**	0.130	**-0.205**		**0.001**	0.086
CSA × CTV / weight	0.053		0.399	**<0.001**	**0.349**	*	**<0.001**	**0.032**	**0.230**	*	**<0.001**	**0.013**	**-0.187**		**0.003**	0.131
CSA × CTV / BMI	**0.248**	** **	**<0.001**	0.184	**0.289**	** **	**<0.001**	0.159	**0.181**	** **	**0.004**	0.057	**-0.144**	** **	**0.022**	0.309
Women	Gripr-value	n=	217p-value	vs. SMIp-value	Sit upr-value	n=	201p-value	vs. SMIp-value	Balancer-value	n=	217p-value	vs. SMIp-value	Reactionr-value	n=	217p-value	vs. SMIp-value
**Quantity**																
CSA	**0.371**		**<0.001**	0.710	0.081		0.254	0.715	0.027		0.689	0.996	-0.016		0.820	0.502
CSA / height	**0.304**		**<0.001**	0.687	0.082		0.248	0.707	0.056		0.410	0.768	-0.028		0.681	0.589
CSA / height^2^	**0.218**		**0.001**	0.172	0.079		0.268	0.734	0.083		0.226	0.569	-0.041		0.553	0.680
CSA / weight	0.032		0.637	**0.001**	**0.312**	*	**<0.001**	**0.006**	**0.213**		**0.002**	0.051	**-0.197**		**0.004**	0.217
CSA / BMI	**0.206**		**0.002**	0.136	**0.305**	*	**<0.001**	**0.007**	**0.152**		**0.026**	0.197	**-0.166**		**0.015**	0.368
ASM	**0.458**		**<0.001**	0.142	-0.029		0.685	0.876	-0.078		0.252	0.601	0.089		0.191	0.925
SMI	**0.339**		**<0.001**	–	-0.045		0.531	–	-0.028		0.684	–	0.080		0.240	–
ASM / weight	**0.172**		**0.011**	0.064	**0.348**	*	**<0.001**	**0.002**	**0.173**		**0.011**	0.129	**-0.151**		**0.027**	0.459
ASM / BMI	**0.359**		**<0.001**	0.817	**0.274**	*	**<0.001**	**0.019**	0.061		0.372	0.731	-0.090		0.189	0.921
**Quality**																
CTV	-0.005		0.947	**<0.001**	**0.279**	*	**<0.001**	**0.016**	0.075		0.270	0.622	-0.103		0.132	0.813
CTV / BMI	**-0.159**		**0.019**	**0.047**	**0.262**	*	**<0.001**	**0.026**	0.117		0.086	0.352	-0.133		0.050	0.579
KES / CSA	**0.192**		**0.005**	0.101	**0.305**	*	**<0.001**	**0.007**	0.054		0.426	0.783	**-0.291**	*	**<0.001**	**0.023**
**Quality× Quantity**																
CSA × CTV	**0.336**		**<0.001**	0.966	**0.188**		**0.008**	0.147	0.064		0.352	0.711	-0.057		0.402	0.812
CSA × CTV / height	**0.269**		**<0.001**	0.427	**0.193**		**0.006**	0.133	0.091		0.184	0.514	-0.071		0.299	0.923
CSA × CTV / height^2^	**0.190**		**0.005**	0.096	**0.190**		**0.007**	0.143	0.113		0.097	0.374	-0.082		0.228	0.983
CSA × CTV / weight	0.023		0.738	**0.001**	**0.350**	*	**<0.001**	**0.001**	**0.208**		**0.002**	0.058	**-0.191**		**0.005**	0.243
CSA × CTV / BMI	**0.168**	** **	**0.014**	0.057	**0.351**	*	**<0.001**	**0.001**	**0.162**	** **	**0.017**	0.162	**-0.169**	** **	**0.013**	0.353

Partial correlation: adjusted for age.

Bold: p<0.05.

* is significantly greater in absolute value than the correlation coefficient between SMI and the same physical function in a test of difference of correlations (p<0.05).

Abbreviations: CSA, cross-sectional area; BMI, body mass index; ASM, appendicular skeletal muscle mass; SMI, skeletal muscle mass index; CTV, computed tomography attenuation value; KES, knee extension strength.

### No correction

In CSA for men, significant correlations were observed for seven variables. In CTV for men, significant correlations were observed for four variables. In CSA×CTV for men, significant correlations were found for seven variables. In CSA for women, significant correlations were observed for three variables. In CTV for women, significant correlations were observed for two variables. In CSA×CTV for women, significant correlations were observed for four variables. Those that showed significantly superior correlations compared to SMI were leg power for men’s CSA (p=0.049), KES for women’s CSA (p=0.001), KES for women’s CSA×CTV (p<0.001), and sit-up for women’s CTV (p=0.016).

### Height correction

KES for men showed significant correlations for CSA (r=0.523), CSA/height (r=0.526), and CSA/height^2^ (r=0.498), while KES for women also showed significant correlations for CSA (r=0.606), CSA/height (r=0.604), and CSA/height^2^ (r=0.570). Leg power for men was significantly correlated with CSA (r=0.422), CSA/height (r=0.355), and CSA/height^2^ (r=0.270), whereas leg power for women was significantly correlated with CSA (r=0.365), CSA/height (r=0.338), and CSA/height^2^ (r=0.293). Correlations were as strong with height correction as without, particularly for KES and leg power.

### Weight correction

In CSA/weight for men, significant correlations were observed for seven variables. In CSA×CTV/weight for men, significant correlations were observed for seven variables. In CSA/weight for women, significant correlations were observed for five variables. In CSA×CTV/weight for women, significant correlations were observed for five variables. Compared with other correction methods, weight correction showed a poorer relationship with the direct function of muscle output, such as strength and power; nevertheless, it was significantly correlated with several other variables. In particular, with a particularly strong relationship between sit-up and balance.

### BMI correction

In CSA/BMI for men, significant correlations were observed for all eight variables. In CSA×CTV/BMI for men, significant correlations were observed for all eight variables. In CSA/BMI for women, significant correlations were observed for all eight variables. In CSA×CTV/BMI for women, significant correlations were observed for seven variables. Correction by BMI was a measurement variable related to most physical functions.

### Diagnostic performance

In the diagnostic accuracy evaluation, when values for both men and women are listed, those of the men are presented first.

### Sarcopenia diagnosis

CSA (AUC 0.935), CSA×CTV (AUC 0.936), CSA/height (AUC 0.917), and CSA×CTV/height (AUC 0.920) showed higher and better diagnostic accuracy than SMI (AUC 0.915) in men; however, no significant difference was detected (p>0.05). CSA (AUC 0.809) and CSA×CTV (AUC 0.824) showed moderate diagnostic accuracy in women, which was significantly inferior to SMI (AUC 0.932; p<0.05). Grip strength (AUC 0.946, 0.976) showed the best diagnostic performance among all variables. ASM (AUC 0.943, 0.904) showed the same high accuracy as SMI (AUC 0.915, 0.932), but the diagnostic performance of other corrections, such as ASM/weight (AUC 0.767, 0.772) and ASM/BMI (AUC 0.873, 0.756), was only moderate (**Table 4**).

**Table 4:** Receiver operating characteristic curve for diagnosis vs. Sarcopenia.

Men								Women								
	AUC	95% CI		Cut off	Specificity	Sensitivity	vs. SMI		AUC		95% CI		Cut off	Specificity	Sensitivity	vs. SMI
		Min	Max								Min	Max				
**Quantity**								**Quantity**								
CSA, cm^2^	**0.935**	0.875	0.994	45.7	0.917	0.857	0.518	CSA, cm^2^	**0.809**		0.680	0.938	36.8	0.776	0.727	0.046
CSA / height, cm	**0.917**	0.863	0.971	0.304	0.842	0.857	0.952	CSA / height, cm	**0.799**		0.675	0.923	0.243	0.761	0.727	0.026
CSA / height^2^, %	**0.871**	0.797	0.944	0.191	0.758	0.857	0.167	CSA / height^2^, %	**0.766**		0.647	0.885	0.166	0.654	0.909	0.005
CSA / weight, cm^2^/kg	**0.731**	0.612	0.850	0.894	0.558	0.857	0.005	CSA / weight, cm^2^/kg	0.637		0.487	0.788	0.714	0.771	0.545	<0.001
CSA / BMI, 10^4^·cm^4^/kg	**0.875**	0.791	0.960	2.21	0.833	0.857	0.388	CSA / BMI, 10^4^·cm^4^/kg	0.690		0.535	0.845	1.76	0.693	0.636	0.001
ASM, kg	**0.943**	0.885	1.000	18.4	0.875	0.929	0.323	ASM, kg	**0.904**		0.832	0.975	12.5	0.834	0.818	0.366
SMI, kg / m^2^	**0.915**	0.868	0.963	6.91	0.812	1.000		SMI, kg / m^2^	**0.932**		0.897	0.967	5.38	0.878	1.000	
ASM / weight, %	**0.767**	0.665	0.868	31.8	0.650	0.857	0.008	ASM / weight, %	**0.772**		0.633	0.911	26.5	0.649	0.818	0.018
ASM / BMI, m^2^	**0.873**	0.776	0.970	0.814	0.821	0.857	0.020	ASM / BMI, m^2^	**0.756**		0.599	0.912	0.586	0.776	0.636	0.398
**Quality**								**Quality**								
CTV, HU	**0.746**	0.620	0.871	53.4	0.654	0.857	0.010	CTV, HU	0.638		0.481	0.794	50.9	0.566	0.818	0.001
CTV / BMI, HU·m^2^/kg	0.583	0.433	0.734	2.40	0.562	0.714	<0.001	CTV / BMI, HU·m^2^/kg	0.576		0.419	0.734	2.49	0.698	0.545	<0.001
KES / CSA, N/cm^2^	0.560	0.406	0.713	7.81	0.487	0.714	<0.001	KES / CSA, N/cm^2^	0.647		0.425	0.869	5.88	0.805	0.636	0.016
**Quality× Quantity**								**Quality× Quantity**								
CSA × CTV, cm^2^·HU	**0.936**	0.882	0.990	2418	0.925	0.857	0.451	CSA × CTV, cm^2^·HU	**0.824**		0.743	0.905	1935	0.673	1.000	0.006
CSA × CTV / height, cm·HU	**0.920**	0.866	0.974	16.0	0.846	0.857	0.867	CSA × CTV / height, cm·HU	**0.814**		0.731	0.897	12.8	0.654	1.000	0.005
CSA × CTV / height^2^, HU	**0.883**	0.813	0.953	0.101	0.783	0.857	0.328	CSA × CTV / height^2^, HU	**0.780**		0.681	0.880	0.084	0.644	0.909	0.004
CSA × CTV / weight, cm^2^·HU/kg	**0.775**	0.676	0.874	47.1	0.646	0.786	0.014	CSA × CTV / weight, cm^2^·HU/kg	0.671		0.542	0.800	37.5	0.668	0.727	<0.001
CSA × CTV / BMI, 10^4^·cm^4^·HU/kg	**0.872**	0.799	0.946	115.1	0.825	0.857	0.305	CSA × CTV / BMI, 10^4^·cm^4^·HU/kg	**0.723**		0.605	0.842	89.6	0.654	0.727	<0.001
**Physical performance**								**Physical performance**								
Grip, kg	**0.946**	0.896	0.996	30.0	0.912	0.857	0.360	Grip, kg	**0.976**	*	0.954	0.999	17.9	0.917	1.000	0.046
KES, N	**0.858**	0.789	0.927	382	0.775	0.857	0.148	KES, N	**0.800**		0.684	0.916	255	0.693	0.727	0.039
Leg Power, W	**0.856**	0.775	0.938	396	0.818	0.786	0.149	Leg Power, W	**0.791**		0.710	0.872	274	0.736	0.900	0.002
Sit up, times	**0.826**	0.727	0.925	10.0	0.792	0.769	0.115	Sit up, times	0.641		0.413	0.869	3.0	0.728	0.667	0.015
Walking, m/seconds	**0.846**	0.757	0.936	1.32	0.738	0.786	0.120	Walking, m/seconds	0.486		0.292	0.679	1.38	0.483	0.545	<0.001
Fast walking, m/seconds	**0.798**	0.673	0.922	1.80	0.750	0.786	0.077	Fast walking, m/seconds	0.607		0.401	0.813	1.77	0.539	0.727	0.002
Balance, seconds	**0.788**	0.652	0.923	4.00	0.733	0.714	0.087	Balance, seconds	**0.707**		0.552	0.861	5.00	0.580	0.727	0.005
Reaction, seconds	**0.724**	0.581	0.866	0.479	0.803	0.643	0.012	Reaction, seconds	0.488		0.323	0.653	0.439	0.405	0.727	<0.001

Age, years	**0.892**	0.816	0.968	72.0	0.821	0.857	0.543	Age, years	**0.735**		0.587	0.882	67.0	0.702	0.727	0.010

**Bold**: 0.700 or higher; **red bold**: 0.900 or higher.

* Significant higher AUC relative to SMI (p<0.05).

Abbreviations: AUC, area under the curve; CI, confidence interval; CSA, cross-sectional area; BMI, body mass index; ASM, appendicular skeletal muscle mass; SMI, skeletal muscle mass index; CTV, computed tomography attenuation value; HU, Hounsfield unit; KES, knee extension strength.

### Low muscle mass diagnosis

CSA (AUC 0.867, 0.791), CSA/height (AUC 0.878, 0.807), CSA/height^2^ (AUC 0.876, 0.805), CSA×CTV (AUC 0.814, 0.737), CSA×CTV/height (AUC 0.829, 0.748), and CSA×CTV/height^2^ (AUC 0.829, 0.748) exhibited moderate accuracy in both men and women and showed significantly better diagnostic accuracy than grip strength (AUC 0.681, 0.717) in men only (p<0.001 for all). In DXA measurements, ASM (AUC 0.904, 0.922) had a high accuracy in both men and women and showed significantly better diagnostic performance than grip strength (p<0.001) (**Table 5**).

**Table 5:** Receiver operating characteristic curve for diagnosis vs. low muscle mass.

Men									Women								
	AUC		95% CI		Cut off	Specificity	Sensitivity	vs. grip		AUC		95% CI		Cut off	Specificity	Sensitivity	vs. grip
			Min	Max								Min	Max				
**Quantity**									**Quantity**								
CSA, cm^2^	**0.867**	*	0.820	0.914	55.2	0.785	0.824	<0.001	CSA, cm^2^	**0.791**		0.720	0.861	38.8	0.729	0.744	0.139
CSA / height, cm	**0.878**	*	0.833	0.923	0.328	0.801	0.824	<0.001	CSA / height, cm	**0.807**		0.734	0.880	0.245	0.814	0.692	0.089
CSA / height^2^, %	**0.876**	*	0.831	0.921	0.201	0.753	0.853	<0.001	CSA / height^2^, %	**0.805**		0.727	0.883	0.16	0.802	0.692	0.120
CSA / weight, cm^2^/kg	0.633		0.561	0.705	0.905	0.554	0.691	0.304	CSA / weight, cm^2^/kg	0.499		0.402	0.595	0.783	0.458	0.641	0.003
CSA / BMI, 10^4^·cm^4^/kg	0.645		0.570	0.720	2.43	0.667	0.603	0.356	CSA / BMI, 10^4^·cm^4^/kg	0.506		0.408	0.603	1.94	0.559	0.564	0.007
ASM, kg	**0.904**	*	0.868	0.941	19.9	0.790	0.882	<0.001	ASM, kg	**0.922**	*	0.885	0.960	13.0	0.836	0.846	<0.001
SMI, kg / m^2^	–		–	–	–	–	–	–	SMI, kg / m^2^	–		–	–	–	–	–	–
ASM / weight, %	0.642		0.565	0.719	31.8	0.699	0.603	0.402	ASM / weight, %	0.587		0.482	0.692	27.0	0.571	0.641	0.014
ASM / BMI, m^2^	0.605		0.524	0.686	0.871	0.656	0.544	0.047	ASM / BMI, m^2^	0.572		0.469	0.674	0.638	0.559	0.590	0.004
**Quality**									**Quality**								
CTV, HU	0.509		0.428	0.589	54.9	0.516	0.529	0.008	CTV, HU	0.465		0.362	0.569	50.9	0.542	0.487	<0.001
CTV / BMI, HU·m^2^/kg	**0.784**		0.721	0.847	2.40	0.667	0.824	0.051	CTV / BMI, HU·m^2^/kg	**0.787**		0.709	0.865	2.43	0.695	0.769	0.315
KES / CSA, N/cm^2^	0.534		0.452	0.615	7.99	0.608	0.485	0.021	KES / CSA, N/cm^2^	0.526		0.426	0.626	6.98	0.508	0.538	<0.001
**Quality× Quantity**									**Quality× Quantity**								
CSA × CTV, cm^2^·HU	**0.814**	*	0.759	0.870	3070	0.720	0.779	<0.001	CSA × CTV, cm^2^·HU	**0.737**		0.662	0.813	2072	0.605	0.821	0.664
CSA × CTV / height, cm·HU	**0.829**	*	0.777	0.880	17.9	0.747	0.765	<0.001	CSA × CTV / height, cm·HU	**0.748**		0.669	0.826	13.0	0.678	0.744	0.541
CSA × CTV / height^2^, HU	**0.829**	*	0.778	0.880	0.107	0.742	0.794	<0.001	CSA × CTV / height^2^, HU	**0.748**		0.666	0.831	0.084	0.689	0.718	0.563
CSA × CTV / weight, cm^2^·HU/kg	0.609		0.536	0.682	50.3	0.548	0.691	0.101	CSA × CTV / weight, cm^2^·HU/kg	0.503		0.406	0.600	41.4	0.497	0.590	<0.001
CSA × CTV / BMI, 10^4^·cm^4^·HU/kg	0.614		0.537	0.691	126.0	0.742	0.485	0.089	CSA × CTV / BMI, 10^4^·cm^4^·HU/kg	0.506		0.410	0.602	99.2	0.475	0.590	<0.001
**Physical performance**									**Physical performance**								
Grip, kg	0.681		0.609	0.753	37.0	0.613	0.632		Grip, kg	**0.717**		0.632	0.802	22.5	0.650	0.667	
KES, N	**0.736**		0.670	0.802	441	0.613	0.735	0.095	KES, N	**0.709**		0.629	0.788	275	0.621	0.744	0.860
Leg Power, W	0.668		0.596	0.740	563	0.497	0.750	0.864	Leg Power, W	**0.701**		0.612	0.790	290	0.687	0.703	0.637
Sit up, times	0.605		0.524	0.685	12.0	0.639	0.530	0.086	Sit up, times	0.465		0.361	0.569	10.0	0.527	0.543	0.002
Walking, m/seconds	0.563		0.481	0.645	1.38	0.565	0.529	0.008	Walking, m/seconds	0.519		0.415	0.623	1.38	0.480	0.513	0.007
Fast walking, m/seconds	0.617		0.542	0.693	1.93	0.516	0.721	0.149	Fast walking, m/seconds	0.499		0.394	0.603	1.78	0.494	0.590	<0.001
Balance, seconds	0.606		0.526	0.686	8.00	0.532	0.662	0.097	Balance, seconds	0.509		0.404	0.614	6.00	0.446	0.615	0.010
Reaction, seconds	0.571		0.492	0.650	0.44	0.659	0.500	0.024	Reaction, seconds	0.519		0.430	0.608	0.47	0.424	0.667	0.003
Age, years	0.632		0.553	0.712	65.0	0.683	0.544	0.211	Age, years	0.496		0.393	0.599	61.0	0.497	0.538	<0.001

**Bold** is 0.700 or higher, **Red bold** is 0.900 or higher.

* Significant higher AUC relative to grip (p<0.05).

Abbreviations: AUC, area under the curve; CI, confidence interval; CSA, cross-sectional area; BMI, body mass index; ASM, appendicular skeletal muscle mass; SMI, skeletal muscle mass index; CTV, computed tomography attenuation value; HU, Hounsfield unit; KES, knee extension strength.

### Low muscle function diagnosis

None of the variables with high accuracy were found in the CT and DXA measurements in both men and women. CSA×CTV (AUC 0.868, 0.810; p=0.039, 0.007) and CSA (AUC 0.853, 0.769; p=0.047, 0.039) were significantly better than SMI (AUC 0.774. 0.643) in both men and women. CSA×CTV/height (AUC 0.782; p=0.022) also showed a significantly better diagnostic performance than SMI in women. ASM (AUC 0.867, 0.768) showed moderate accuracy and significantly better diagnostic performance than SMI in both men and women (p=0.006, p<0.001), whereas ASM/BMI (AUC 0.883, 0.740) showed moderate accuracy and was significantly better than SMI in men (p=0.035) (**Table 6**).

**Table 6:** Receiver operating characteristic curve for diagnosis vs. low muscle function.

Men									Women								
	AUC		95% CI		Cut off	Specificity	Sensitivity	vs. SMI		AUC		95% CI		Cut off	Specificity	Sensitivity	vs. SMI
			Min	Max								Min	Max				
**Quantity**									**Quantity**								
CSA, cm^2^	**0.853**	*	0.779	0.927	51.1	0.838	0.760	0.047	CSA, cm^2^	**0.769**	*	0.682	0.856	39.1	0.692	0.742	0.039
CSA / height, cm	**0.817**		0.738	0.895	0.327	0.694	0.800	0.273	CSA / height, cm	**0.727**		0.636	0.818	0.258	0.659	0.710	0.145
CSA / height^2^, %	**0.752**		0.659	0.844	0.203	0.607	0.760	0.590	CSA / height^2^, %	0.663		0.567	0.759	0.167	0.665	0.645	0.708
CSA / weight, cm^2^/kg	**0.713**		0.603	0.824	0.853	0.721	0.600	0.417	CSA / weight, cm^2^/kg	0.647		0.542	0.752	0.765	0.665	0.613	0.960
CSA / BMI, 10^4^·cm^4^/kg	**0.848**		0.768	0.927	2.29	0.803	0.760	0.200	CSA / BMI, 10^4^·cm^4^/kg	**0.730**		0.633	0.828	1.62	0.859	0.548	0.244
ASM, kg	**0.867**	*	0.793	0.942	19.2	0.769	0.880	0.006	ASM, kg	**0.768**	*	0.694	0.841	14.0	0.600	0.871	<0.001
SMI, kg / m^2^	**0.774**		0.690	0.858	7.18	0.699	0.800	–	SMI, kg / m^2^	0.643		0.539	0.747	5.86	0.600	0.645	–
ASM / weight, %	**0.791**		0.708	0.875	31.8	0.672	0.840	0.788	ASM / weight, %	0.671		0.559	0.784	26.8	0.622	0.710	0.636
ASM / BMI, m^2^	**0.883**	*	0.821	0.946	0.814	0.847	0.800	0.035	ASM / BMI, m^2^	**0.740**		0.639	0.842	0.622	0.692	0.677	0.149
**Quality**									**Quality**								
CTV, HU	**0.759**		0.657	0.861	53.4	0.677	0.840	0.830	CTV, HU	**0.720**		0.625	0.814	50.9	0.605	0.806	0.320
CTV / BMI, HU·m^2^/kg	0.559		0.434	0.685	2.20	0.686	0.440	0.026	CTV / BMI, HU·m^2^/kg	0.584		0.478	0.691	2.29	0.530	0.581	0.517
KES / CSA, N/cm^2^	**0.703**		0.591	0.815	7.25	0.716	0.680	0.412	KES / CSA, N/cm^2^	0.686		0.573	0.798	6.27	0.757	0.613	0.609
**Quality× Quantity**									**Quality× Quantity**								
CSA × CTV, cm^2^·HU	**0.868**	*	0.791	0.945	2678	0.821	0.760	0.039	CSA × CTV, cm^2^·HU	**0.810**	*	0.731	0.889	1935	0.719	0.839	0.007
CSA × CTV / height, cm·HU	**0.842**		0.762	0.923	17.4	0.716	0.880	0.137	CSA × CTV / height, cm·HU	**0.782**	*	0.698	0.865	12.8	0.692	0.806	0.022
CSA × CTV / height^2^, HU	**0.794**		0.705	0.883	0.108	0.629	0.880	0.680	CSA × CTV / height^2^, HU	**0.737**		0.647	0.827	0.0843	0.676	0.742	0.116
CSA × CTV / weight, cm^2^·HU/kg	**0.759**		0.661	0.856	47.2	0.664	0.760	0.824	CSA × CTV / weight, cm^2^·HU/kg	**0.702**		0.606	0.798	37.7	0.697	0.710	0.424
CSA × CTV / BMI, 10^4^·cm^4^·HU/kg	**0.850**		0.774	0.926	115	0.843	0.720	0.180	CSA × CTV / BMI, 10^4^·cm^4^·HU/kg	**0.767**		0.681	0.853	89.6	0.697	0.742	0.084
**Physical performance**									**Physical performance**								
Grip, kg	**0.980**	*	0.955	1.000	30.0	0.956	0.920	<0.001	Grip, kg	**0.978**	*	0.948	1.000	17.9	1.000	0.903	<0.001
KES, N	**0.871**	*	0.812	0.931	382	0.799	0.800	0.049	KES, N	**0.816**	*	0.748	0.885	255	0.735	0.710	0.007
Leg Power, W	**0.888**		0.834	0.943	446	0.769	0.917	0.069	Leg Power, W	**0.777**	*	0.704	0.850	278	0.737	0.714	0.040
Sit up, times	**0.838**		0.745	0.931	10.0	0.819	0.783	0.296	Sit up, times	0.692		0.579	0.805	8.00	0.611	0.760	0.732
Walking, m/seconds	**0.852**		0.767	0.937	1.32	0.764	0.800	0.205	Walking, m/seconds	0.648		0.537	0.760	1.33	0.627	0.613	0.952
Fast walking, m/seconds	**0.841**		0.747	0.934	1.80	0.777	0.800	0.333	Fast walking, m/seconds	**0.739**		0.634	0.845	1.67	0.795	0.633	0.277
Balance, seconds	**0.772**		0.678	0.865	5.00	0.672	0.720	0.969	Balance, seconds	**0.794**	*	0.717	0.871	5.00	0.632	0.839	0.045
Reaction, seconds	**0.784**		0.693	0.875	0.479	0.829	0.680	0.877	Reaction, seconds	0.643		0.535	0.751	0.493	0.724	0.516	1.000
Age, years	**0.915**	*	0.86	0.971	72.0	0.856	0.88	0.007	Age, years	**0.828**	*	0.748	0.907	67.0	0.757	0.774	0.011

**Bold** is 0.700 or higher, **Red bold** is 0.900 or higher.

* Significant higher AUC relative to SMI (p<0.05).

Abbreviations: AUC, area under the curve; CI, confidence interval; CSA, cross-sectional area; BMI, body mass index; ASM, appendicular skeletal muscle mass; SMI, skeletal muscle mass index; CTV, computed tomography attenuation value; HU, Hounsfield unit; KES, knee extension strength.

### Validation of diagnostic performance in an external validation cohort

The characteristics of the participants are summarized in (**Table 7**) **Table 8**. As for sarcopenia, CSA (DOR 5.55, 19.86), CSA/height (DOR 5.23, 14.45), and CSA/height^2^ (DOR 5.57, 11.21) were good. CSA×CTV (DOR 6.05) was favorable in men, whereas CSA×CTV had a high DOR (29.82) but much lower accuracy (0.39) in women. As for low muscle mass, men displayed good diagnostic performance with respect to CSA (DOR 9.89), CSA×CTV (DOR 11.71), CSA/height (DOR 12.64), CSA/height^2^ (DOR 14.15), CSA×CTV/height (DOR 9.18), and CSA×CTV/height^2^ (DOR 13.41). Women had better CSA (DOR 23.61), CSA/height (DOR 14.32), and CSA/height^2^ (DOR 13.77); CSA×CTV and its height-corrected values had a higher DOR, but lower accuracy. As for low muscle function, CSA (DOR 3.28, 3.86), CSA×CTV (DOR 5.23, 5.20), CSA/BMI (DOR 4.93, 5.42), and CSA×CTV/BMI (DOR 4.54, 6.51) showed good diagnostic performance. Women also had good CTV (DOR 5.14) and CSA×CTV/weight (DOR 4.81).

**Table 7:** Validation of diagnostic performance in the external validation cohort.

Men	Sarcopenia				Low muscle mass				Low muscle function			
	Specificity	Sensitivity	Accuracy	DOR	Specificity	Sensitivity	Accuracy	DOR	Specificity	Sensitivity	Accuracy	DOR
CSA	0.75	0.66	0.72	5.55	0.48	0.92	0.76	9.89	0.46	0.80	0.62	3.28
CSA/height	0.55	0.82	0.64	5.23	0.54	0.92	0.79	12.64	0.33	0.82	0.56	2.12
CSA/height^2^	0.53	0.84	0.63	5.57	0.60	0.91	0.80	14.15	0.30	0.77	0.53	1.42
CSA/weight	0.23	0.91	0.45	2.74	0.21	0.87	0.64	1.81	0.34	0.80	0.56	2.05
CSA/BMI	0.37	0.84	0.53	3.00	0.23	0.85	0.63	1.69	0.39	0.89	0.63	4.93
ASM	0.51	0.96	0.65	17.37	0.46	0.99	0.80	48.96	0.36	0.88	0.61	3.79
SMI	0.57	0.96	0.70	22.59	–	–	–	–	0.31	0.74	0.52	1.29
ASM/weight	0.17	0.82	0.38	0.87	0.17	0.83	0.59	0.98	0.17	0.83	0.49	1.01
ASM/BMI	0.36	0.75	0.49	1.67	0.19	0.84	0.61	1.22	0.44	0.80	0.62	3.10
CTV	0.11	0.96	0.39	2.19	0.06	0.98	0.65	2.63	0.13	0.95	0.53	2.76
CTV/BMI	0.20	0.75	0.38	0.73	0.00	0.67	0.43	0.02	0.47	0.68	0.57	1.85
KES/CSA	0.07	0.98	0.36	2.20	0.02	0.95	0.62	0.59	0.16	0.95	0.54	3.45
CSA*CTV	0.51	0.86	0.62	6.05	0.25	0.98	0.72	11.71	0.40	0.89	0.64	5.23
CSA*CTV/height	0.41	0.91	0.57	6.19	0.27	0.97	0.72	9.18	0.23	0.94	0.57	4.14
CSA*CTV/height^2^	0.33	0.91	0.52	4.46	0.35	0.97	0.75	13.41	0.20	0.92	0.55	2.82
CSA*CTV/weight	0.19	0.93	0.43	2.79	0.08	0.94	0.64	1.52	0.21	0.92	0.56	3.07
CSA*CTV/BMI	0.33	0.86	0.50	2.94	0.17	0.85	0.61	1.16	0.40	0.88	0.63	4.54
Women	Sarcopenia				Low muscle mass				Low muscle function			
	Specificity	Sensitivity	Accuracy	DOR	Specificity	Sensitivity	Accuracy	DOR	Specificity	Sensitivity	Accuracy	DOR
CSA	0.55	0.95	0.62	19.86	0.47	0.97	0.62	23.61	0.47	0.81	0.65	3.86
CSA/height	0.56	0.93	0.63	14.45	0.60	0.91	0.69	14.32	0.47	0.75	0.61	2.52
CSA/height^2^	0.49	0.93	0.57	11.21	0.66	0.88	0.72	13.77	0.47	0.66	0.57	1.75
CSA/weight	0.47	0.58	0.49	1.23	0.30	0.75	0.44	1.30	0.43	0.78	0.61	2.64
CSA/BMI	0.30	0.84	0.40	2.11	0.18	0.90	0.39	1.83	0.63	0.76	0.70	5.42
ASM	0.76	0.95	0.79	51.23	0.74	0.94	0.80	41.61	0.39	0.75	0.57	1.92
SMI	0.87	0.98	0.89	182.78	–	–	–	–	0.52	0.53	0.52	1.19
ASM/weight	0.40	0.77	0.47	2.10	0.31	0.74	0.44	1.27	0.40	0.76	0.58	2.08
ASM/BMI	0.49	0.70	0.53	2.14	0.22	0.83	0.40	1.27	0.45	0.89	0.67	6.34
CTV	0.10	1.00	0.26	9.38	0.09	0.96	0.35	1.98	0.13	0.98	0.56	5.14
CTV/BMI	0.10	0.77	0.22	0.36	0.07	0.64	0.24	0.13	0.27	0.86	0.57	2.19
KES/CSA	0.21	0.91	0.34	2.38	0.07	0.96	0.33	1.43	0.19	0.94	0.57	3.62
CSA*CTV	0.25	1.00	0.39	29.82	0.19	0.99	0.43	10.86	0.33	0.92	0.63	5.20
CSA*CTV/height	0.27	1.00	0.40	31.49	0.26	0.99	0.48	16.49	0.33	0.89	0.61	3.78
CSA*CTV/height^2^	0.29	1.00	0.42	34.99	0.33	0.99	0.52	22.11	0.30	0.86	0.59	2.66
CSA*CTV/weight	0.27	0.88	0.38	2.53	0.17	0.90	0.38	1.68	0.36	0.90	0.63	4.81
CSA*CTV/BMI	0.25	0.93	0.38	3.86	0.12	0.91	0.35	1.32	0.36	0.92	0.65	6.51

Abbreviations: DOR, diagnostic odds ratio; CSA, cross-sectional area; BMI, body mass index; ASM, appendicular skeletal muscle mass; SMI, skeletal muscle mass index; CTV, computed tomography attenuation value; HU, Hounsfield unit; KES, knee extension strength

**Table 8:** Characteristics of the external validation cohort.

	Men		Women		P value
	n=135		n=232		
Age, years	78.0	±6.4	76.8	±7.4	0.117
Body height, cm	162.3	±6.2	149.3	±6.5	<0.001
Body weight, kg	60.8	±10.0	51.5	±10.1	<0.001
BMI, kg/m^2^	23.1	±3.4	23.1	±4.2	0.977
**Sarcopenia diagnosis**					
Low muscle mass, n(%)	87	(64.4)	69	(29.7)	<0.001
Low muscle function, n(%)	65	(48.1)	118	(50.9)	0.665
Sarcopenia, n(%)	44	(32.6)	43	(18.5)	0.003
**Quantity**					
CSA, cm^2^	48.0	±9.7	36.2	±6.9	<0.001
CSA / Height, cm	0.295	±0.056	0.242	±0.043	<0.001
CSA / Height^2^, %	0.182	±0.034	0.162	±0.029	<0.001
CSA / Weight, cm^2^/kg	0.791	±0.115	0.713	±0.127	<0.001
CSA / BMI, 10^4^·cm^4^/kg	2.09	±0.34	1.59	±0.32	<0.001
ASM, kg	17.7	±2.7	13.1	±2.3	<0.001
SMI, kg / m^2^	6.70	±0.84	5.88	±0.91	<0.001
ASM / Weight, %	29.3	±2.6	25.7	±2.6	<0.001
ASM / BMI, m^2^	0.77	±0.10	0.58	±0.08	<0.001
**Quality**					
CTV, HU	46.8	±5.6	43.5	±5.7	<0.001
CTV / BMI, HU·m^2^/kg	2.08	±0.41	1.96	±0.46	0.01
KES / CSA, N/cm^2^	5.41	±1.49	4.87	±1.34	<0.001
**Quality×Quantity**					
CSA × CTV, cm^2^·HU	2266	±606	1587	±421	<0.001
CSA × CTV / Height, cm·HU	13.9	±3.5	10.6	±2.7	<0.001
CSA × CTV / Height^2^, HU	0.086	±0.021	0.071	±0.017	<0.001
CSA × CTV / Weight, cm^2^·HU/kg	37.4	±8.5	31.4	±8.3	<0.001
CSA × CTV / BMI, 10^4^·cm^4^·HU/kg	99	±25	70	±20	<0.001
**Physical performance**					
Grip, kg	30.0	±6.9	20.9	±5.3	<0.001
KES, N	259	±88	176	±60	<0.001

Values are expressed as number (%).

Mean ± standard deviation.

Mean ± standard deviation.

P values were obtained using the t-test for continuous data and the χ^2^ test and fisher's exact test for categorical data.

Abbreviations: BMI, body mass index; CSA, cross-sectional area; ASM, appendicular skeletal muscle mass; SMI, skeletal muscle mass index; CTV, computed tomography attenuation value; HU, Hounsfield unit; KES, knee extension strength.

## Discussion

This study is the first to compare physical function, muscle mass, and muscle quality-related parameters measured using CT and DXA, including various corrections, and investigate the relationship between muscle mass loss, muscle function loss, and sarcopenia diagnosis based on the AWGS 2019 diagnostic criteria in the general Asian population.

Parameters such as CSA, CTV, and CSA×CTV, which can be calculated using CT, showed a correlation with many physical functions. As the correlations with physical functions vary depending on the parameters and correction methods, it is important to consider using different parameters for different purposes. Because of the small difference, no direct statistical testing was performed. However, the uncorrected CSA×CTV, examined as a composite item of muscle mass and quality, showed a slightly stronger association than the uncorrected CSA in many functions. This tendency was stronger in men than in women, suggesting that CSA×CTV may be a better indicator of overall physical function than CSA or CTV alone. We previously reported that KES was independently related to Qc CSA and CTV (14), in the present study, the Qc muscle is the primary working muscle for KES and power; hence, uncorrected CSA, CSA×CTV, and height-corrected variables are suitable for evaluation. Regarding height correction, there was almost no difference in correction by height^2^. Considering the possibility of overcorrection, we believe that correction by height is sufficient. In functions other than muscle strength, weight correction for CSA, CSA×CTV, and ASM showed several correlations, indicating that weight correction is a good index for evaluating motor abilities other than muscle strength. In sit-ups, weight-corrected CSA, CSA×CTV, and ASM showed better correlation than other correction methods in both men and women, indicating a high degree of specificity. Although CTV is not highly correlated with physical function, it was most highly correlated in endurance events such as sit-ups. CTV reflects fatty infiltration, and the lower value of CTV when fat is abundant is thought to be a major factor influencing CTV reduction in muscles (27). Part of CTV may be derived from type 1 fibers, which have a high-fat content in myocytes (28, 29). This indicates that the presence of more type 1 fibers in the muscle would decrease the CTV; however, the present results indicated a positive correlation, which is the opposite of that assumed to be derived from muscle fiber type. The reasons for this may include that the effect of fat infiltration may be more significant than the effect of muscle fiber type and that the rectus femoris is the only Qc muscle directly involved in upper body raising and the trunk muscle is the main working muscle, which may not have been directly evaluated.

The correction for BMI is also noteworthy. ASM/BMI is most strongly and directly correlated with weakness and slowness (10), and dividing the muscle mass by BMI is a good method for physical function assessment. In CT measurements, the correction for BMI showed significant correlations with several physical functions. Indeed, CSA/BMI was significantly correlated with all physical functions in both sexes, and CSA×CTV/BMI was significantly correlated with all physical functions, except for normal walking speed in women. Correcting indices that include muscle mass, such as CSA and CSA×CTV, with BMI is considered a good index for objective evaluation of a wide range of physical functions other than muscle strength in both sexes. Qualitative changes in muscle quality have been reported to result in fatty infiltration in skeletal muscles (5, 30) and changes in the proportion of muscle fibers (31, 32), leading to muscle weakness and decreased physical performance (33–35). However, physical performance can be objectively evaluated using indices that consider muscle mass, muscle quality, and obesity; this partially supports previous reports that muscle strength and physical performance cannot necessarily be evaluated based on muscle mass alone. In measurements using CT, the simultaneous evaluation of fat content and muscle mass may be used as a suitable index for sarcopenia, which was previously assessed based on the combined evaluation of muscle mass loss and decline in muscle strength and physical function. The relationship between qualitative muscle changes and muscle function is thought to be based on the relationship between intramuscular adipose tissue and visceral fat, while visceral fat is strongly correlated with muscle mass loss and ectopic fat, such as intramuscular adipose tissue (36, 37). In particular, fatty infiltration is likely to increase in patients with diabetes and non-alcoholic fatty liver (38–40). While the relationship of muscle quality and quantity with motor function was clarified in this study, the long-term prognosis remains unclear. However, based on reports on the association between CTV and life-threatening diseases, the CTV in muscle assessment may be a major surrogate marker that can predict life expectancy. Further studies on this topic are warranted.

Regarding the diagnostic accuracy of sarcopenia, CSA, CSA×CTV, and their correction by height were highly accurate in men, showing a diagnostic accuracy comparable to that of ASM and SMI, which reflects the total body muscle mass. However, we found little difference between CSA and CSA×CTV. In men, the Qc muscle mass or its correction for height alone was highly indicative of sarcopenia, which was diagnosed using SMI and grip strength or walking speed. Hence, the assessment of Qc muscle mass is quite meaningful, and CSA×CTV is an excellent criterion; nonetheless, its measurement method is challenging compared with that of CSA alone. CSA and CSA×CTV showed moderate diagnostic accuracy in women, which was lower than that in men. This suggests a slight difference between the indicated CT measurements of Qc muscle mass and SMI in women. A minor improvement in the accuracy of sarcopenia diagnosis was observed by using CSA×CTV. Although no statistical comparisons were made, the external validation cohort was an older cohort with lower muscle mass and strength and a higher prevalence of sarcopenia than the internal cohort. Validation using the cutoffs determined for the internal cohort showed that measurements that were good AUCs in the internal cohort were more likely to show good DOR in the external cohort but with some differences in diagnostic accuracy. Taking into account the external validation cohort results, it is still concise to use CSA or CSA height correction, but the use of CSA×CTV and height correction may be considered for higher sensitivity.

Regarding the relationship with muscle mass, the correction of CSA and CSA×CTV by height and height^2^ showed good diagnostic accuracy in men, although not as robust as that of ASM. These parameters showed good diagnostic value in women but were not as good as in men. This sex difference may be related to the fundamental question of whether the SMI is appropriate as a reference value for sarcopenia, especially in women. Indeed, Baumgartner et al. reported that the relationship of SMI with physical function and frailty in women was relatively weaker when they first proposed SMI as ASM/height^2^ for the diagnosis of sarcopenia and muscle mass loss (41). In a previously reported analysis of our cohort, the decline in height with age was greater in women than in men, and the non-apparent decline in SMI in women is thought to be due to the greater decline in height, as compared with the decline in muscle mass with aging (11, 42). It could be inferred that some women have “hidden muscle mass loss,” a condition in which the SMI does not decrease but the muscle mass decreases. However, given that the difference in the diagnosis of sarcopenia and muscle mass loss is greater than that of ASM without height correction, the Qc muscle in women may not be representative of total body muscle mass. As we only included middle-aged and older people in the present study, it was impossible to determine the clear criteria for muscle mass loss, such as the -2 standard deviation in younger people. Additionally, because this was a cross-sectional study, the longitudinal significance of these parameters could not be evaluated. Therefore, it was not possible to determine whether Qc muscle analysis is an appropriate method for assessing those who cannot be evaluated well using SMI. Future studies in this regard are warranted. Taking into account the external validation cohort results, the use of CSA or CSA height and height^2^ correction is concise for the diagnosis of muscle mass loss based on SMI; however, the use of CSA×CTV and its height and height^2^ correction may also be considered for higher sensitivity.

With respect to physical function, CSA, CSA×CTV, ASM, and their BMI correction, as well as height correction in CSA and CSA×CTV, showed good diagnostic accuracy, with similar accuracies in both men and women. Thus, an increase in diagnostic performance could be expected when CSA and CTV are evaluated simultaneously. The accuracy of CT and DXA measurements in assessing muscle function is similar to that of KES, and they more accurately reflect motor function decline than SMI. This could be because these indices are more closely related to physical function than SMI, as previously mentioned. Furthermore, ASM and ASM/BMI are more likely to be associated with prognosis than SMI (11). Although prognoses were not assessed in this study, CSA, CSA×CTV, and their BMI-corrected values may be assessed with both physical function and prognosis. Future studies must evaluate their long-term association. Taking into account the external validation cohort, it seems advisable to consider the use of CSA or its BMI correction or, with the expectation of high sensitivity, CSA×CTV or its BMI correction for muscle dysfunction.

The present study has some limitations. First, this was a cross-sectional study, and a longitudinal study is therefore required to clarify the effect of each measurement. Second, CT has disadvantages such as radiation exposure, cost, and equipment requirements. Nevertheless, it is not necessary to perform CT on the entire length of the Qc muscle, and radiation exposure can, therefore, be minimized. Third, the CTV is difficult to use as an absolute value because there may be racial differences in this parameter (30).

For CT to be widely used for muscle evaluation in the future, it is necessary to verify the differences between models and to automate a more objective and simple measurement method. This is because the possibility that performance differences between models and differences in image processing may affect measurement values cannot be denied. It would be necessary, for example, to evaluate differences between models by photographing the same subject and verifying the differences. Nonetheless, we believe that a detailed Qc muscle evaluation using CT with relative objectivity, as in the present study, can be used to confirm the validity and reproducibility of the reference values and to evaluate non-invasive techniques such as echoes, which have a narrower imaging range and tend to be less reproducible and can also contribute to the development of other modalities. The external validation cohort was used; however, this cohort was older than the base cohort, which may have resulted in differences in sensitivity and specificity due to spectrum bias.

In conclusion, we investigated the relationship of Qc muscle mass and muscle quality with physical function and its use in diagnosing sarcopenia. In both men and women, Qc CT measurements, represented by CSA, showed a good association with motor function, and simultaneous assessment of CSA and CTV and BMI correction increased the correlation with several motor functions. Sarcopenia diagnosis based on SMI, CSA, and CSA×CTV and their correction by height were highly accurate and useful for diagnosis in men but were only moderately accurate in women. Overall, these results suggest that CT imaging of the Qc muscle is a useful diagnostic method for sarcopenic changes such as muscle mass loss, muscle weakness, and loss of muscle function.

## Data availability statement

The data analyzed in this study is subject to the following licenses/restrictions: Not available to outsiders. Requests to access these datasets should be directed to RO, otsuka@ncgg.go.jp.

## Ethics statement

The studies involving humans were approved by The Ethics and Conflict of Interest Committee of the National Center for Geriatrics and Gerontology. The studies were conducted in accordance with the local legislation and institutional requirements. The participants provided their written informed consent to participate in this study.

## Author contributions

TM: Formal analysis, Validation, Visualization, Writing – original draft. YM: Conceptualization, Funding acquisition, Writing – review & editing. MTo: Formal analysis, Writing – review & editing. YS: Software, Writing – review & editing. SIs: Supervision, Writing – review & editing. TW: Methodology, Writing – review & editing. MTa: Validation, Writing – review & editing. YN: Data curation, Investigation, Writing – review & editing. CT: Data curation, Formal Analysis, Writing – review & editing. HS: Funding acquisition, Supervision, Writing – review & editing. SIm: Supervision, Writing – review & editing. RO: Investigation, Project administration, Resources, Writing – review & editing. HA: Supervision, Writing – review & editing.

## References

[1] Beaudart C Zaaria M Pasleau F Reginster J-Y Bruyère O. Health outcomes of sarcopenia: A systematic review and meta-analysis. PloS One (2017) 12:e0169548. doi: 10.1371/journal.pone.0169548 28095426 PMC5240970

[2] Cruz-Jentoft AJ Bahat G Bauer J Boirie Y Bruyère O Cederholm T. Sarcopenia: Revised European consensus on definition and diagnosis. Age Ageing (2019) 48:16–31. doi: 10.1093/ageing/afy169 30312372 PMC6322506

[3] Yamada M Kimura Y Ishiyama D Nishio N Abe Y Kakehi T. Differential characteristics of skeletal muscle in community-dwelling older adults. J Am Med Dir Assoc (2017) 18:807.e9–807.e16. doi: 10.1016/j.jamda.2017.05.011 28676289

[4] Visser M Goodpaster BH Kritchevsky SB Newman AB Nevitt M Rubin SM. Muscle mass, muscle strength, and muscle fat infiltration as predictors of incident mobility limitations in well-functioning older persons. J Gerontol A Biol Sci Med Sci (2005) 60:324–33. doi: 10.1093/gerona/60.3.324 15860469

[5] Azzabou N Hogrel JY Carlier PG. NMR based biomarkers to study age-related changes in the human quadriceps. Exp Gerontol (2015) 70:54–60. doi: 10.1016/j.exger.2015.06.015 26122131

[6] Sergi G Trevisan C Veronese N Lucato P Manzato E. Imaging of sarcopenia. Eur J Radiol (2016) 85:1519–24. doi: 10.1016/j.ejrad.2016.04.009 27117135

[7] Giovannini S Brau F Forino R Berti A D’ignazio F Loreti C. Sarcopenia: Diagnosis and management, state of the art and contribution of ultrasound. J Clin Med (2021) 10:5552. doi: 10.3390/jcm10235552 34884255 PMC8658070

[8] Goodpaster BH Thaete FL Kelley DE. Composition of skeletal muscle evaluated with computed tomography. Ann N Y Acad Sci (2000) 904:18–24. doi: 10.1111/j.1749-6632.2000.tb06416.x 10865705

[9] Anderson DE D’Agostino JM Bruno AG Demissie S Kiel DP Bouxsein ML. Variations of CT-based trunk muscle attenuation by age, sex, and specific muscle. J Gerontol A Biol Sci Med Sci (2013) 68:317–23. doi: 10.1093/gerona/gls168 PMC3605905 22904095

[10] Cawthon PM Peters KW Shardell MD McLean RR Dam TTL Kenny AM. Cutpoints for low appendicular lean mass that identify older adults with clinically significant weakness. J Gerontol A Biol Sci Med Sci (2014) 69:567–75. doi: 10.1093/gerona/glu023 PMC3991141 24737559

[11] Otsuka R Matsui Y Tange C Nishita Y Tomida M Ando F. What is the best adjustment of appendicular lean mass for predicting mortality or disability among Japanese community dwellers? BMC Geriatr (2018) 18:8. doi: 10.1186/s12877-017-0699-6 29304751 PMC5756439

[12] Kim KM Jang HC Lim S. Differences among skeletal muscle mass indices derived from height-, weight-, and body mass index-adjusted models in assessing sarcopenia. Korean J Intern Med (2016) 31:643–50. doi: 10.3904/kjim.2016.015 PMC4939509 27334763

[13] Kasai T Ishiguro N Matsui Y Harada A Takemura M Yuki A. Sex- and age-related differences in mid-thigh composition and muscle quality determined by computed tomography in middle-aged and elderly Japanese. Geriatr Gerontol Int (2015) 15:700–6. doi: 10.1111/ggi.12338 25244543

[14] Mizuno T Matsui Y Tomida M Suzuki Y Nishita Y Tange C. Differences in the mass and quality of the quadriceps with age and sex and their relationships with knee extension strength. J Cachexia Sarcopenia Muscle (2021) 12:900–12. doi: 10.1002/jcsm.12715 PMC8350198 34009738

[15] Naruse M Trappe S Trappe TA. Human skeletal muscle-specific atrophy with aging: a comprehensive review. J Appl Physiol (2023) 134:900–14. doi: 10.1152/japplphysiol.00768.2022 PMC10069966 36825643

[16] Oba H Matsui Y Arai H Watanabe T Iida H Mizuno T. Evaluation of muscle quality and quantity for the assessment of sarcopenia using mid-thigh computed tomography: a cohort study. BMC Geriatr (2021) 21:239. doi: 10.1186/s12877-021-02187-w 33849469 PMC8045267

[17] Shimokata H Ando F Niino N. A new comprehensive study on aging-the national institute for longevity sciences, longitudinal study of aging (NILS-LSA). J Epidemiol (2000) 10:1–9. doi: 10.2188/jea.10.1sup_1 10835822

[18] Hirano Y Yamada Y Matsui Y Ota S Arai H. Lower limb muscle quality and phase angle contribute to the reduced walking speed among older adults. Geriatr Gerontol Int (2022) 22:603–9. doi: 10.1111/ggi.14423 35781752

[19] Kuriyama K Matsui Y Suzuki Y Mizuno T Watanabe T Takemura M. Relationship between sarcopenia classification and thigh muscle mass, fat area, muscle CT value and osteoporosis in middle-aged and older Japanese adults. Bone (2022) 163:116487. doi: 10.1016/j.bone.2022.116487 35843483

[20] Tracy BL Ivey FM Hurlbut D Martel GF Lemmer JT Siegel EL. Muscle quality. II. Effects of strength training in 65- to 75-yr-old men and women. J Appl Physiol (1999) 86:195–201. doi: 10.1152/jappl.1999.86.1.195 9887131

[21] Fujita R Matsui Y Harada A Takemura M Kondo I Nemoto T. Does the Q – H index show a stronger relationship than the H:Q ratio in regard to knee pain during daily activities in patients with knee osteoarthritis? J Phys Ther Sci (2016) 28:3320–4. doi: 10.1589/jpts.28.3320 PMC5276753 28174444

[22] Meng X Rosenthal R Rubin DB. Comparing correlated correlation coefficients. Psychol Bull (1992) 111:172–5. doi: 10.1037/0033-2909.111.1.172

[23] Chen LK Woo J Assantachai P Auyeung TW Chou MY Iijima K. Asian working group for sarcopenia: 2019 consensus update on sarcopenia diagnosis and treatment. J Am Med Dir Assoc (2020) 21:300–307.e2. doi: 10.1016/j.jamda.2019.12.012 32033882

[24] DeLong ER DeLong DM Clarke-Pearson DL. Comparing the areas under two or more correlated receiver operating characteristic curves: A nonparametric approach. Biometrics (1988) 44:837–45. doi: 10.2307/2531595 3203132

[25] Moses LE Shapiro D Littenberg B. Combining independent studies of a diagnostic test into a summary roc curve: Data-analytic approaches and some additional considerations. Stat Med (1993) 12:1293–316. doi: 10.1002/sim.4780121403 8210827

[26] Glas AS Lijmer JG Prins MH Bonsel GJ Bossuyt PMM. The diagnostic odds ratio: A single indicator of test performance. J Clin Epidemiol (2003) 56:1129–35. doi: 10.1016/S0895-4356(03)00177-X 14615004

[27] Goodpaster BH Kelley DE Thaete FL He J Ross R. Skeletal muscle attenuation determined by computed tomography is associated with skeletal muscle lipid content. J Appl Physiol (2000) 89:104–10. doi: 10.1152/jappl.2000.89.1.104 10904041

[28] Schrauwen-Hinderling VB Hesselink MKC Schrauwen P Kooi ME. Intramyocellular lipid content in human skeletal muscle. Obesity (2006) 14:357–67. doi: 10.1038/oby.2006.47 16648604

[29] Staron RS Hagerman FC Hikida RS Murray TF Hostler DP Crill MT. Fiber type composition of the vastus lateralis muscle of young men and women. J Histochem Cytochem (2000) 48:623–9. doi: 10.1177/002215540004800506 10769046

[30] Goodpaster BH Carlson CL Visser M Kelley DE Scherzinger A Harris TB. Attenuation of skeletal muscle and strength in the elderly: The health ABC study. J Appl Physiol (2001) 90:2157–65. doi: 10.1152/jappl.2001.90.6.2157 11356778

[31] Lexell J Taylor CC Sjöström M. What is the cause of the ageing atrophy? Total number, size and proportion of different fiber types studied in whole vastus lateralis muscle from 15- to 83-year-old men. J Neurol Sci (1988) 84:275–94. doi: 10.1016/0022-510X(88)90132-3 3379447

[32] Mattiello-Sverzut AC Chimelli L Moura MS de A Teixeira S de Oliveira JAM. The effects of aging on biceps brachii muscle fibers: a morphometrical study from biopsies and autopsies. Arq Neuropsiquiatr (2003) 61:555–60. doi: 10.1590/S0004-282X2003000400006 14513157

[33] Young H-J Jenkins NT Zhao Q Mccully KK. Measurement of intramuscular fat by muscle echo intensity. Muscle Nerve (2015) 52:963–71. doi: 10.1002/mus.24656 PMC4575231 25787260

[34] Akima H Yoshiko A Tomita A Ando R Saito A Ogawa M. Relationship between quadriceps echo intensity and functional and morphological characteristics in older men and women. Arch Gerontol Geriatr (2017) 70:105–11. doi: 10.1016/j.archger.2017.01.014 28126635

[35] Watanabe Y Yamada Y Fukumoto Y Ishihara T Yokoyama K Yoshida T. Echo intensity obtained from ultrasonography images reflecting muscle strength in elderly men. Clin Interv Aging (2013) 8:993–8. doi: 10.2147/CIA.S47263 PMC3732157 23926426

[36] Lim S Meigs JB. Ectopic fat and cardiometabolic and vascular risk. Int J Cardiol (2013) 169:166–76. doi: 10.1016/j.ijcard.2013.08.077 24063931

[37] Yang YX Chong MS Lim WS Tay L Yew S Yeo A. Validity of estimating muscle and fat volume from a single MRI section in older adults with sarcopenia and sarcopenic obesity. Clin Radiol (2017) 72:427.e9–427.e14. doi: 10.1016/j.crad.2016.12.011 28117037

[38] Park SW Goodpaster BH Strotmeyer ES de Rekeneire N Harris TB Schwartz AV. Decreased muscle strength and quality in older adults with type 2 diabetes: the health, aging, and body composition study. Diabetes (2006) 55:1813–8. doi: 10.2337/db05-1183 16731847

[39] Komiya H Mori Y Yokose T Kurokawa N Horie N Tajima N. Effect of intramuscular fat difference on glucose and insulin reaction in oral glucose tolerance test. J Atheroscler Thromb (2006) 13:136–42. doi: 10.5551/jat.13.136 16835468

[40] Kitajima Y Hyogo H Sumida Y Eguchi Y Ono N Kuwashiro T. Severity of non-alcoholic steatohepatitis is associated with substitution of adipose tissue in skeletal muscle. J Gastroenterol Hepatol (2013) 28:1507–14. doi: 10.1111/jgh.12227 23577962

[41] Baumgartner RN Koehler KM Gallagher D Romero L Heymsfield SB Ross RR. Epidemiology of sarcopenia among the elderly in New Mexico. Am J Epidemiol (1998) 147:755–63. doi: 10.1093/oxfordjournals.aje.a009520 9554417

[42] OtsukaRNishitaYTangeCTomidaMKatoYImaiT. Age-related 12-year changes in dietary diversity and food intakes among community-dwelling Japanese aged 40 to 79 years. J Nutr Health Aging (2018) 22:594–600. doi: 10.1007/s12603-018-0999-3 29717759

