# Supplementary material for: Relationship between quadriceps muscle computed tomography measurement and motor function, muscle mass, and sarcopenia diagnosis
Source: Front Endocrinol (Lausanne). 2023 Nov 16;14:1259350. doi: 10.3389/fendo.2023.1259350 (PMC10693452; doi:10.3389/fendo.2023.1259350)
Supplement: Supplementary file 1 [file Table_1.docx]

| Table S1. Correlation of physical functions with computed tomography and dual-energy X-ray absorptiometry measurement (1) | | | | | | | | | | | | | | | | | | | |  |
| --- | --- | --- | --- | --- | --- | --- | --- | --- | --- | --- | --- | --- | --- | --- | --- | --- | --- | --- | --- | --- |
| **Men** | **KES** | n= | 254 | vs. SMI |  | **Leg power** | n= | 245 | vs. SMI |  | **Walking** | n= | 254 | vs. SMI |  | **Fast walking** | n= | 254 | vs. SMI |  |
|  | r-value |  | p-value | p-value |  | r-value |  | p-value | p-value |  | r-value |  | p-value | p-value |  | r-value |  | p-value | p-value |  |
| **Quantity** |  |  |  |  |  |  |  |  |  |  |  |  |  |  |  |  |  |  |  |  |
| CSA | **0.659** | ***** | **<0.001** | 0.001 |  | **0.597** | ***** | **<0.001** | <0.001 |  | **0.365** | ***** | **<0.001** | 0.019 |  | **0.385** | ***** | **<0.001** | 0.034 |  |
| CSA / Height | **0.636** | ***** | **<0.001** | 0.003 |  | **0.518** | ***** | **<0.001** | 0.013 |  | **0.338** | ***** | **<0.001** | 0.046 |  | **0.351** |  | **<0.001** | 0.093 |  |
| CSA / Height^2^ | **0.574** |  | **<0.001** | 0.055 |  | **0.403** |  | **<0.001** | 0.381 |  | **0.288** |  | **<0.001** | 0.166 |  | **0.294** |  | **<0.001** | 0.334 |  |
| CSA / Weight | **0.513** |  | **<0.001** | 0.349 |  | **0.364** |  | **<0.001** | 0.702 |  | **0.339** | ***** | **<0.001** | 0.044 |  | **0.351** |  | **<0.001** | 0.094 |  |
| CSA / BMI | **0.594** | ***** | **<0.001** | 0.024 |  | **0.574** | ***** | **<0.001** | 0.001 |  | **0.415** | ***** | **<0.001** | 0.003 |  | **0.440** | ***** | **<0.001** | 0.004 |  |
| ASM | **0.496** |  | **<0.001** | 0.496 |  | **0.529** | ***** | **<0.001** | 0.008 |  | **0.255** |  | **<0.001** | 0.329 |  | **0.309** |  | **<0.001** | 0.250 |  |
| SMI | **0.448** |  | **<0.001** | - |  | **0.334** |  | **<0.001** | - |  | **0.172** |  | **0.006** | - |  | **0.213** |  | **<0.001** | - |  |
| ASM / Weight | **0.331** |  | **0.006** | 0.119 |  | **0.270** |  | **<0.001** | 0.437 |  | **0.227** |  | **<0.001** | 0.516 |  | **0.293** |  | **<0.001** | 0.340 |  |
| ASM / BMI | **0.374** |  | **0.047** | 0.317 |  | **0.477** |  | **<0.001** | 0.059 |  | **0.287** |  | **<0.001** | 0.173 |  | **0.349** |  | **<0.001** | 0.098 |  |
| **Quality** |  |  |  |  |  |  |  |  |  |  |  |  |  |  |  |  |  |  |  |  |
| CTV | **0.398** |  | **<0.001** | 0.494 |  | **0.351** |  | **<0.001** | 0.832 |  | **0.384** | ***** | **<0.001** | 0.009 |  | **0.324** |  | **<0.001** | 0.180 |  |
| CTV / BMI | -0.003 |  | 0.961 | <0.001 |  | 0.035 |  | 0.587 | 0.001 |  | **0.144** |  | **0.021** | 0.755 |  | 0.121 |  | 0.055 | 0.286 |  |
| KES / CSA | **0.728** | ***** | **<0.001** | <0.001 |  | **0.371** |  | **<0.001** | 0.637 |  | **0.236** |  | **<0.001** | 0.448 |  | **0.215** |  | **<0.001** | 0.979 |  |
| **Quality×Quantity** |  |  |  |  |  |  |  |  |  |  |  |  |  |  |  |  |  |  |  |  |
| CSA × CTV | **0.696** | ***** | **<0.001** | <0.001 |  | **0.625** | ***** | **<0.001** | <0.001 |  | **0.431** | ***** | **<0.001** | 0.001 |  | **0.431** | ***** | **<0.001** | 0.006 |  |
| CSA × CTV / Height | **0.685** | ***** | **<0.001** | <0.001 |  | **0.563** | ***** | **<0.001** | 0.001 |  | **0.415** | ***** | **<0.001** | 0.003 |  | **0.408** | ***** | **<0.001** | 0.015 |  |
| CSA × CTV / Height^2^ | **0.641** | ***** | **<0.001** | 0.002 |  | **0.471** |  | **<0.001** | 0.070 |  | **0.378** | ***** | **<0.001** | 0.012 |  | **0.365** |  | **<0.001** | 0.062 |  |
| CSA × CTV / Weight | **0.539** |  | **<0.001** | 0.181 |  | **0.403** |  | **<0.001** | 0.378 |  | **0.393** | ***** | **<0.001** | 0.007 |  | **0.383** | ***** | **<0.001** | 0.036 |  |
| CSA × CTV / BMI | **0.588** | ***** | **<0.001** | 0.031 |  | **0.555** | ***** | **<0.001** | 0.002 |  | **0.440** | ***** | **<0.001** | 0.001 |  | **0.442** | ***** | **<0.001** | 0.004 |  |
| **Women** | **KES** | n= | 217 | vs. SMI |  | **Leg power** | n= | 204 | vs. SMI |  | **Walking** | n= | 216 | vs. SMI |  | **Fast walking** | n= | 215 | vs. SMI |  |
|  | r-value |  | p-value | p-value |  | r-value |  | p-value | p-value |  | r-value |  | p-value | p-value |  | r-value |  | p-value | p-value |  |
| **Quantity** |  |  |  |  |  |  |  |  |  |  |  |  |  |  |  |  |  |  |  |  |
| CSA | **0.673** | ***** | **<0.001** | <0.001 |  | **0.473** | ***** | **<0.001** | 0.005 |  | **0.195** |  | **0.004** | 0.109 |  | **0.296** | ***** | **<0.001** | 0.003 |  |
| CSA / Height | **0.663** | ***** | **<0.001** | <0.001 |  | **0.430** | ***** | **<0.001** | 0.023 |  | **0.147** |  | **0.031** | 0.279 |  | **0.236** | ***** | **<0.001** | 0.024 |  |
| CSA / Height^2^ | **0.613** | ***** | **<0.001** | <0.001 |  | **0.358** |  | **<0.001** | 0.157 |  | 0.081 |  | 0.234 | 0.689 |  | **0.152** |  | **0.026** | 0.174 |  |
| CSA / Weight | **0.403** |  | **<0.001** | 0.522 |  | **0.223** |  | **0.001** | 0.947 |  | **0.188** |  | **0.006** | 0.129 |  | **0.308** | ***** | **<0.001** | 0.002 |  |
| CSA / BMI | **0.484** |  | **<0.001** | 0.092 |  | **0.350** |  | **<0.001** | 0.186 |  | **0.290** | ***** | **<0.001** | 0.008 |  | **0.435** | ***** | **<0.001** | <0.001 |  |
| ASM | **0.439** |  | **<0.001** | 0.272 |  | **0.370** |  | **<0.001** | 0.122 |  | **0.174** |  | **0.010** | 0.170 |  | **0.169** |  | **0.013** | 0.124 |  |
| SMI | **0.350** |  | **<0.001** | - |  | **0.229** |  | **0.001** | - |  | 0.043 |  | 0.532 | - |  | -0.021 |  | 0.758 | - |  |
| ASM / Weight | **0.149** |  | **0.028** | 0.026 |  | 0.120 |  | 0.086 | 0.259 |  | **0.231** | ***** | **<0.001** | 0.048 |  | **0.223** | ***** | **0.001** | 0.034 |  |
| ASM / BMI | **0.292** |  | **<0.001** | 0.509 |  | **0.302** |  | **<0.001** | 0.432 |  | **0.338** | ***** | **<0.001** | 0.001 |  | **0.391** | ***** | **<0.001** | <0.001 |  |
| **Quality** |  |  |  |  |  |  |  |  |  |  |  |  |  |  |  |  |  |  |  |  |
| CTV | **0.320** |  | **<0.001** | 0.726 |  | **0.247** |  | **<0.001** | 0.853 |  | **0.145** |  | **0.033** | 0.286 |  | **0.315** | ***** | **<0.001** | 0.002 |  |
| CTV / BMI | -0.039 |  | 0.570 | 0.001 |  | -0.004 |  | 0.954 | 0.021 |  | **0.152** |  | **0.025** | 0.254 |  | **0.267** | ***** | **<0.001** | 0.009 |  |
| KES / CSA | **0.727** | ***** | **<0.001** | <0.001 |  | **0.333** |  | **<0.001** | 0.258 |  | **0.197** |  | **0.004** | 0.107 |  | **0.299** | ***** | **<0.001** | 0.003 |  |
| **Quality×Quantity** |  |  |  |  |  |  |  |  |  |  |  |  |  |  |  |  |  |  |  |  |
| CSA × CTV | **0.690** | ***** | **<0.001** | <0.001 |  | **0.498** | ***** | **<0.001** | 0.002 |  | **0.213** |  | **0.002** | 0.072 |  | **0.366** | ***** | **<0.001** | <0.001 |  |
| CSA × CTV / Height | **0.681** | ***** | **<0.001** | <0.001 |  | **0.464** | ***** | **<0.001** | 0.007 |  | **0.175** |  | **0.010** | 0.166 |  | **0.322** | ***** | **<0.001** | 0.001 |  |
| CSA × CTV / Height^2^ | **0.644** | ***** | **<0.001** | <0.001 |  | **0.407** | ***** | **<0.001** | 0.046 |  | 0.125 |  | 0.068 | 0.395 |  | **0.259** | ***** | **<0.001** | 0.012 |  |
| CSA × CTV / Weight | **0.423** |  | **<0.001** | 0.371 |  | **0.259** |  | **<0.001** | 0.753 |  | **0.197** |  | **0.004** | 0.105 |  | **0.355** | ***** | **<0.001** | <0.001 |  |
| CSA × CTV / BMI | **0.487** |  | **<0.001** | 0.085 |  | **0.357** |  | **<0.001** | 0.160 |  | **0.276** | ***** | **<0.001** | 0.013 |  | **0.451** | ***** | **<0.001** | <0.001 |  |
| Bold: p < 0.05  * is significantly greater in absolute value than the correlation coefficient between SMI and the same physical function in a test of difference of correlations (p<0.05). | | | | | | | | | | | | | | | | | | | | |
| Abbreviations: CSA, cross-sectional area; BMI, body mass index; ASM, appendicular skeletal muscle mass; SMI, skeletal muscle mass index; CTV, computed tomography attenuation value; KES, knee extension strength | | | | | | | | | | | | | | | | | | | |  |

| Table S2. Correlation of physical functions and computed tomography with dual-energy X-ray absorptiometry measurement (2) | | | | | | | | | | | | | | | | | | | |  |
| --- | --- | --- | --- | --- | --- | --- | --- | --- | --- | --- | --- | --- | --- | --- | --- | --- | --- | --- | --- | --- |
| **Men** | **Grip** | n= | 254 | vs. SMI |  | **Sit-up** | n= | 249 | vs. SMI |  | **Balance** | n= | 254 | vs. SMI |  | **Reaction** | n= | 253 | vs. SMI |  |
|  | r-value |  | p-value | p-value |  | r-value |  | p-value | p-value |  | r-value |  | p-value | p-value |  | r-value |  | p-value | p-value |  |
| **Quantity** |  |  |  |  |  |  |  |  |  |  |  |  |  |  |  |  |  |  |  |  |
| CSA | **0.555** | ***** | **<0.001** | 0.027 |  | **0.427** | ***** | **<0.001** | 0.024 |  | **0.225** |  | **<0.001** | 0.057 |  | **-0.328** | ***** | **<0.001** | 0.024 |  |
| CSA / Height | **0.457** |  | **<0.001** | 0.456 |  | **0.408** | ***** | **<0.001** | 0.046 |  | **0.225** |  | **<0.001** | 0.057 |  | **-0.321** | ***** | **<0.001** | 0.029 |  |
| CSA / Height^2^ | **0.327** |  | **<0.001** | 0.324 |  | **0.364** |  | **<0.001** | 0.156 |  | **0.210** |  | **<0.001** | 0.083 |  | **-0.295** |  | **<0.001** | 0.063 |  |
| CSA / Weight | **0.268** |  | **<0.001** | 0.087 |  | **0.491** | ***** | **<0.001** | 0.002 |  | **0.334** | ***** | **<0.001** | 0.001 |  | **-0.334** | ***** | **<0.001** | 0.019 |  |
| CSA / BMI | **0.531** |  | **<0.001** | 0.066 |  | **0.537** | ***** | **<0.001** | <0.001 |  | **0.333** | ***** | **<0.001** | 0.001 |  | **-0.361** | ***** | **<0.001** | 0.007 |  |
| ASM | **0.622** | ***** | **<0.001** | 0.001 |  | **0.303** |  | **<0.001** | 0.507 |  | 0.079 |  | 0.210 | 0.821 |  | **-0.172** |  | **0.006** | 0.695 |  |
| SMI | **0.403** |  | **<0.001** | - |  | **0.248** |  | **<0.001** | - |  | 0.059 |  | 0.351 | - |  | **-0.137** |  | **0.029** | - |  |
| ASM / Weight | **0.372** |  | **<0.001** | 0.679 |  | **0.454** | ***** | **<0.001** | 0.008 |  | **0.223** |  | **<0.001** | 0.061 |  | **-0.177** |  | **0.005** | 0.650 |  |
| ASM / BMI | **0.598** | ***** | **<0.001** | 0.003 |  | **0.407** | ***** | **<0.001** | 0.047 |  | **0.177** |  | **0.005** | 0.179 |  | **-0.189** |  | **0.003** | 0.551 |  |
| **Quality** |  |  |  |  |  |  |  |  |  |  |  |  |  |  |  |  |  |  |  |  |
| CTV | **0.342** |  | **<0.001** | 0.425 |  | **0.466** | ***** | **<0.001** | 0.005 |  | **0.252** |  | **<0.001** | 0.026 |  | **-0.316** | ***** | **<0.001** | 0.035 |  |
| CTV / BMI | 0.036 |  | 0.564 | <0.001 |  | **0.230** |  | **<0.001** | 0.831 |  | **0.169** |  | **0.007** | 0.212 |  | **-0.126** |  | **0.046** | 0.892 |  |
| KES / CSA | **0.288** |  | **<0.001** | 0.142 |  | **0.308** |  | **<0.001** | 0.465 |  | **0.172** |  | **0.006** | 0.197 |  | **-0.233** |  | **<0.001** | 0.270 |  |
| **Quality×Quantity** |  |  |  |  |  |  |  |  |  |  |  |  |  |  |  |  |  |  |  |  |
| CSA × CTV | **0.580** | ***** | **<0.001** | 0.008 |  | **0.511** | ***** | **<0.001** | 0.001 |  | **0.279** | ***** | **<0.001** | 0.011 |  | **-0.377** | ***** | **<0.001** | 0.004 |  |
| CSA × CTV / Height | **0.503** |  | **<0.001** | 0.161 |  | **0.505** | ***** | **<0.001** | 0.001 |  | **0.284** | ***** | **<0.001** | 0.009 |  | **-0.377** | ***** | **<0.001** | 0.004 |  |
| CSA × CTV / Height^2^ | **0.396** |  | **<0.001** | 0.927 |  | **0.475** | ***** | **<0.001** | 0.003 |  | **0.277** | ***** | **<0.001** | 0.011 |  | **-0.361** | ***** | **<0.001** | 0.007 |  |
| CSA × CTV / Weight | **0.325** |  | **<0.001** | 0.311 |  | **0.542** | ***** | **<0.001** | <0.001 |  | **0.358** | ***** | **<0.001** | <0.001 |  | **-0.364** | ***** | **<0.001** | 0.007 |  |
| CSA × CTV / BMI | **0.518** |  | **<0.001** | 0.103 |  | **0.561** | ***** | **<0.001** | <0.001 |  | **0.346** | ***** | **<0.001** | 0.001 |  | **-0.374** | ***** | **<0.001** | 0.004 |  |
| **Women** | **Grip** | n= | 217 | vs. SMI |  | **Sit-up** | n= | 201 | vs. SMI |  | **Balance** | n= | 217 | vs. SMI |  | **Reaction** | n= | 217 | vs. SMI |  |
|  | r-value |  | p-value | p-value |  | r-value |  | p-value | p-value |  | r-value |  | p-value | p-value |  | r-value |  | p-value | p-value |  |
| **Quantity** |  |  |  |  |  |  |  |  |  |  |  |  |  |  |  |  |  |  |  |  |
| CSA | **0.504** | ***** | **<0.001** | 0.023 |  | **0.265** | ***** | **<0.001** | 0.012 |  | **0.222** | ***** | **<0.001** | 0.021 |  | **-0.148** |  | **0.029** | 0.365 |  |
| CSA / Height | **0.427** |  | **<0.001** | 0.210 |  | **0.237** | ***** | **<0.001** | 0.026 |  | **0.216** | ***** | **0.001** | 0.025 |  | **-0.137** |  | **0.043** | 0.426 |  |
| CSA / Height^2^ | **0.314** |  | **<0.001** | 0.922 |  | **0.192** |  | **0.006** | 0.079 |  | **0.195** | ***** | **0.004** | 0.044 |  | -0.118 |  | 0.081 | 0.551 |  |
| CSA / Weight | **0.187** |  | **0.006** | 0.132 |  | **0.408** | ***** | **<0.001** | <0.001 |  | **0.331** | ***** | **<0.001** | <0.001 |  | **-0.276** | ***** | **<0.001** | 0.022 |  |
| CSA / BMI | **0.396** |  | **<0.001** | 0.384 |  | **0.458** | ***** | **<0.001** | <0.001 |  | **0.343** | ***** | **<0.001** | <0.001 |  | **-0.287** | ***** | **<0.001** | 0.015 |  |
| ASM | **0.520** | ***** | **<0.001** | 0.013 |  | **0.096** |  | **0.177** | 0.435 |  | 0.056 |  | 0.414 | 0.583 |  | -0.003 |  | 0.970 | 0.543 |  |
| SMI | **0.323** |  | **<0.001** | - |  | -0.018 |  | 0.804 | - |  | -0.003 |  | 0.968 | - |  | 0.061 |  | 0.369 | - |  |
| ASM / Weight | **0.218** |  | **0.001** | 0.244 |  | **0.362** | ***** | **<0.001** | <0.001 |  | **0.217** | ***** | **0.001** | 0.024 |  | **-0.186** |  | **0.006** | 0.189 |  |
| ASM / BMI | **0.479** |  | **<0.001** | 0.053 |  | **0.397** | ***** | **<0.001** | <0.001 |  | **0.227** | ***** | **<0.001** | 0.018 |  | **-0.199** |  | **0.003** | 0.145 |  |
| **Quality** |  |  |  |  |  |  |  |  |  |  |  |  |  |  |  |  |  |  |  |  |
| CTV | **0.220** |  | **0.001** | 0.252 |  | **0.423** | ***** | **<0.001** | <0.001 |  | **0.272** | ***** | **<0.001** | 0.004 |  | **-0.221** |  | **0.001** | 0.090 |  |
| CTV / BMI | 0.003 |  | 0.969 | 0.001 |  | **0.347** | ***** | **<0.001** | 0.001 |  | **0.230** | ***** | **<0.001** | 0.017 |  | **-0.205** |  | **0.002** | 0.128 |  |
| KES / CSA | **0.232** |  | **<0.001** | 0.307 |  | **0.326** | ***** | **<0.001** | 0.001 |  | 0.109 |  | 0.110 | 0.272 |  | **-0.315** | ***** | **<0.001** | 0.006 |  |
| **Quality×Quantity** |  |  |  |  |  |  |  |  |  |  |  |  |  |  |  |  |  |  |  |  |
| CSA × CTV | **0.506** | ***** | **<0.001** | 0.022 |  | **0.386** | ***** | **<0.001** | <0.001 |  | **0.295** | ***** | **<0.001** | 0.002 |  | **-0.210** |  | **0.002** | 0.117 |  |
| CSA × CTV / Height | **0.440** |  | **<0.001** | 0.154 |  | **0.372** | ***** | **<0.001** | <0.001 |  | **0.294** | ***** | **<0.001** | 0.002 |  | **-0.206** |  | **0.002** | 0.126 |  |
| CSA × CTV / Height^2^ | **0.350** |  | **<0.001** | 0.751 |  | **0.342** | ***** | **<0.001** | 0.001 |  | **0.281** | ***** | **<0.001** | 0.003 |  | **-0.195** |  | **0.004** | 0.160 |  |
| CSA × CTV / Weight | **0.228** |  | **<0.001** | 0.289 |  | **0.472** | ***** | **<0.001** | <0.001 |  | **0.365** | ***** | **<0.001** | <0.001 |  | **-0.292** | ***** | **<0.001** | 0.013 |  |
| CSA × CTV / BMI | **0.390** |  | **<0.001** | 0.425 |  | **0.507** | ***** | **<0.001** | <0.001 |  | **0.373** | ***** | **<0.001** | <0.001 |  | **-0.300** | ***** | **<0.001** | 0.010 |  |
| Bold: p < 0.05  * is significantly greater in absolute value than the correlation coefficient between SMI and the same physical function in a test of difference of correlations (p<0.05). | | | | | | | | | | | | | | | | | | | | |
| Abbreviations: CSA, cross-sectional area; BMI, body mass index; ASM, appendicular skeletal muscle mass; SMI, skeletal muscle mass index; CTV, computed tomography attenuation value; KES, knee extension strength | | | | | | | | | | | | | | | | | | | |  |
